# Epidemiology of multiple sclerosis in Iran: A systematic review and meta-analysis

**DOI:** 10.1371/journal.pone.0214738

**Published:** 2019-04-09

**Authors:** Milad Azami, Mohammad Hossein YektaKooshali, Masoumeh Shohani, Ali Khorshidi, Leily Mahmudi

**Affiliations:** 1 Student Research Committee, School of Medicine, Ilam University of Medical Sciences, Ilam, Iran; 2 Student Research Committee, School of Nursing, Midwifery and Paramedicine, Guilan University of Medical Sciences, Rasht, Iran; 3 Department of Nursing, Faculty of Allied Medical Sciences, Ilam University of Medical Sciences, Ilam, Iran; 4 Department of Epidemiology, Faculty of Medicine, Ilam University of Medical Sciences, Ilam, Iran; 5 Faculty of Medicine, Dezful University of Medical Sciences, Dezful, Iran; University of Ioannina School of Medicine, GREECE

## Abstract

**Background:**

Multiple sclerosis (MS) is one of the most common neurological disorders and is one of the main causes of disability. The prevalence and incidence of MS in Iran is reported to range from 5.3 to 89/ 100,000and 7 to 148.1/ 100,000, respectively. There are no systematic and meta-analysis studies on MS in Iran. Therefore, this study was conducted to investigate the prevalence and incidence of MS in Iran using meta-analysis.

**Method:**

A systematic review of the present study focused on MS epidemiology in Iran based on PRISMA guidelines for systematic review and meta-analysis. We searched eight international databases including Scopus, PubMed, Science Direct, Cochrane Library, Web of Science, EMBASE, PsycINFO, Google Scholar search engine and six Persian databases for peer-reviewed studies published without time limit until May 2018. Data were analyzed using Comprehensive meta-analysis ver. 2 software. The review protocol has been registered in PROSPERO with ID: CRD42018114491.

**Results:**

According to searching on different databases, 39 (15%) articles finalized. The prevalence of MS in Iran was estimated 29.3/ 100,000 (95%CI: 25.6–33.5) based on random effects model. The prevalence of MS in men and women was estimated to be 16.5/ 100,000 (95%CI: 13.7–23.4) and 44.8/ 100,000 (95%CI: 36.3–61.6), respectively. The incidence of MS in Iran was estimated to be 3.4/ 100,000 (95%CI: 1.8–6.2) based on random effects model. The incidence of MS in men was estimated to be 16.5/ 100,000 (95%CI: 13.7–23.4) and the incidence of MS in women was 44.8/ 100,000 (95%CI: 36.3–61.6). The meta-regression model for prevalence and incidence of MS was significantly higher in terms of year of study (p<0.001).

**Conclusions:**

The results of this study can provide a general picture of MS epidemiology in Iran. The current meta-analysis showed that the prevalence and incidence of MS in Iran is high and is rising over time.

## 1. Introduction

Multiple sclerosis (MS) is a neurodegenerative and immune-mediated demyelinating disease of the human central nervous system[1–4]. The clinical manifestations of MS include opiate neuritis, central paralysis, sensory imbalance, balance disorder, cognitive impairment, fatigue and sleep disorders[5]. Women are approximately 2–3 times more likely to suffer from MS[6], and most patients are 20 to 50 years old. Residents of Eastern Europe are more likely to suffer from MS compared with residents of Asia, Africa and Latin America[7, 8].

Iran is muslim country in the Middle East with a latitude of 32°00 and a longitude of 53°00 and has 31 provinces. There are various ethnic groups in Iran, including Fars, Kurds, Mazani, Gilak, Lor, Turks, Arabs, and Baluch, and are now united by Iranian culture. According to the World Health Organization (WHO) in 2008, around 1.3 million people had MS worldwide, while in 2013, the prevalence of MS was 73 per 100,000 in the world and was 60 per 100,000 in Iran[9, 10]. At the moment, Iran is well known for its high prevalence of MS in the world, whereas 15 years ago, it was assumed based on the MS slope hypothesis that Iran could be a low-risk area for MS with an incidence of less than 5 per 100,000 people[11–13].

Despite numerous studies, the main cause of MS is still unknown. According to a hypothesis, MS carries out an autoimmune attack against self-myelin or oligodendrocytic antigens by macrophages, deadly T cells, Lymphokines, and antibodies when they enter the brain[14]. A combination of genetic and environmental factors such as latitude, vitamin D use, skin color, migration, meal, smoking, occupational exposure to toxins, stress, or even recent studies of specific viral infections such as Epstein-Barr virus (EBV)[15] and bacterial infections like mycoplasma pneumonia [16, 17] may affect this disease[6, 15–19].

Basic epidemiological information helps to quickly identify, diagnose and control the disease complications[20]. One of the most important goals of meta-analysis, which results from the combination of existing studies, is to increase the volume of samples and the number of studies, to reduce the difference between the available parameters and the confidence interval, which ultimately leads to solving a problem, especially in the field of medicine. In fact, such studies are a vital link between research studies and decision-making at the bedside or policies[21–23].

The prevalence of MS in Iran has been reported to be 5.3–89 per 100,000[2, 5, 10, 11, 13, 21, 22, 24–46]. Considering the above-mentioned issues, controversy in the prevalence of MS, the lack of global access to the precise prevalence of MS in Iran, as well as expressing the final conclusion for policy making and operational planning in Iran, this study was conducted to estimate the prevalence and incidence of MS in Iran by systematically reviewing all available documentations and their combination through meta-analysis.

## 2. Materials and methods

### 2.1. Study protocol

The present systematic review focused on MS epidemiology in Iran based on PRISMA guidelines [47](S1 File) for systematic review and meta-analysis. All the steps of research, including search, selection of studies, qualitative assessment, and data extraction were done independently by two researchers. The agreement was reached by group discussion. The protocol of this review registered at: International Prospective Register of Systematic Reviews(PROSPERO) (https://www.crd.york.ac.uk/PROSPERO/) Identifier: CRD42018114491 [48, 49](S2 File).

### 2.2. Search strategy

The search was performed by two researchers independently. We searched the titles and abstracts of articles in six Persian databases including Scientific Information Database (SID) (http://www.sid.ir/), Barakat Knowledge Network System (http://health.barakatkns.com), (Iranian Research Institute for Information Science and Technology (IranDoc) (https://irandoc.ac.ir), Regional Information Center for Science and Technology (RICST) (http://en.ricest.ac.ir/), Magiran (http://www.magiran.com/), Iranian National Library (http://www.nlai.ir/) and eight international databases including Scopus, PubMed/Medline, Science Direct, Cochrane Library, Web of Science, Embase, PsycINFO as well as Google Scholar search engine for peer-reviewed studies published without time limit until May 2018. The keywords used were 'incidence', 'prevalence', 'epidemiology', 'MS', 'multiple sclerosis' and 'Iran'. Boolean operators (AND & OR) were used to search by a combination of words. A sample of search strategy in PubMed database is shown in Appendix 1. The list of references of the studies was searched manually for additional reports.

### 2.3. Inclusion criteria (PICO)

Inclusion criteria according to **PICO** (Problem or Population, Interventions, Comparison and Outcome) [50, 51]: (1) **P**opulation: all Iranian population, in all age ranges and both genders; (2) **I**ntervention: diagnosis of MS by Poser or McDonald criteria for confirmed MS; (3) **C**omparison: variable aimed for incidence and prevalence of MS such as gender, province, year of study and etc; (4) **O**utcome: Estimate the prevalence and incidence of MS.

### 2.4. Exclusion criteria

The inclusion criteria were all epidemiological studies on MS. The exclusion criteria included: 1. non-random sample size; 2. sample size other than Iranian population; 3. Articles published in languages other than Persian and English; 4. Not relevant to the subject; 5. qualitative studies; case report; review articles, case reports and interventional studies, and 6. duplicate articles.

### 2.5. Quality assessment

Researchers assessed the quality of the selected articles using a scoring system based on the 8-item the modified Newcastle Ottawa Scale (NOS) for non-randomized studies [52] (S2 File). Each question was given a score between 0 and 1. Points 0–5, 6–7 and 8–9 were considered low quality, moderate quality and high quality, respectively. The minimum score for entering the quantitative meta-analysis process was 5 and the articles that acquired the minimum qualitative assessment score entered the process of data extraction and meta-analysis.

### 2.6. Screening and data extraction

Two independent researchers (Azami M, YektaKooshali MH) screened all the articles retrieved by the search strategy based on title and abstract for eligibility according to inclusion and exclusion criteria. Any contradiction between the two researchers was discussed and finally, a consensus was reached. In addition, if necessary, the full text was examined further for more clarification at this stage. In the next step, the researchers were provided with the full text of eligible articles. Each qualified full text was reviewed independently by two researchers and a third expert (Expert-epidemiologist) was there to provide consultations on disagreements between the two researchers.

Data extraction was done by the researchers using a pre-prepared form. The data for the study included the first author, year of publication, year of study, study setting, location, sample size, geographical area, province, the prevalence of MS and MS diagnostic method, which was extracted independently by two researchers and blinded to the author's name, institute, and journal. If necessity, further information, and raw data were requested by contacting the author (first author, corresponding author or contacting the authors' department) (Table 1).

**Table 1:** Characteristics of studies into the meta-analysis.

Ref.	First author, Published Year *	Year of Study	Study Type	Place	Diagnostic criteria	Sample size
[33]	Etemadifar M, 2006	2004–5	Cross-sectional	Isfahan	McDonald	3923255
[67]	Sahraian MA, 2010	1999–2010	Cross-sectional	Tehran	McDonald	13422366
[55]	Elhami SR, 2011	1989–2009	Population based	Tehran	Poser (up to 2001) McDonald	14103853
[36]	Heydarpour P, 2013	1991–2011	Population based	Tehran	McDonald	14103853
[45]	Saadatnia M, 2007	2003–6	Cross-sectional	Isfahan	McDonald	3923255
[34]	Etemadifar M, 2010	2003–2010	Cross-sectional	Isfahan	McDonald	4804458
[59]	Ghandehari K, 2010	2009	Population based	RazaviKhorasan	McDonald	5593079
[59]	Ghandehari K, 2010	2009	Population based	North Khorasan	McDonald	811572
[59]	Ghandehari K, 2010	2009	Population based	Southern Khorasan	McDonald	636420
[2]	Abedini M, 2008	2007	Cross-sectional	Mazandaran	McDonald	2893087
[35]	Hashemilar M, 2011	2005–9	Population based	East Azerbaijan	McDonald	3724620
[40]	Moghtaderi A, 2012	1996–2006	Cross-sectional	Sistan and Balouchestan	McDonald	1346367
[46]	Sharafaddinzadeh N, 2012	1997–2009	Cross-sectional	Khuzestan	McDonald	4200000
[31]	Etemadifar M, 2013	2003–2013	Population based	Isfahan	McDonald	4879312
[62]	Kalanie H, 2003	1996–2001	Cross-sectional	Tehran	Poser	
[26]	Jajvandian R, 2011	2005–2011	Population based	North Khorasan	-	867727
[54]	Ebrahimi HA, 2013	2013	Population based	Kerman	-	2947346
[39]	Moghaddam AH, 2013	2013	Population based	Kerman	McDonald	207192
[68]	Saman-Nezhad B,2012	2012	Population based	Kermanshah	-	851405
[25]	Majdinasab N, 2012	2005–2011	Population based	Khuzestan	McDonald	4531720
[43]	Rezaali S, 2013	2011	Population based	Qom	Poser (up to 2001) and McDonald	1151672
[61]	Izadi S, 2015	2013	Population based	Fars	-	4551718
[53]	Ashtari F, 2011	2007	Cross-sectional	Isfahan		
[37]	Maghzi A, 2010	2003–2007	Population based	Isfahan	McDonald’s criteria and 2005 revisions	4559256
[32]	Etemadifar M, 2014 (Isfahan)	2006–2013	Population based	Isfahan	NR	4879312
[32]	Etemadifar M, 2014 (Tehran)	2006–2013	Population based	Tehran	NR	12183391
[32]	Etemadifar M, 2014 (Fars)	2006–2013	Population based	Fars	NR	4596658
[32]	Etemadifar M, 2014 (Alborz)	2006–2013	Population based	Alborz	NR	2412513
[32]	Etemadifar M, 2014 (Markazi)	2006–2013	Population based	Markazi	NR	1413959
[32]	Etemadifar M, 2014 (ChaharMahaa)	2006–2013	Population based	ChaharMahaal and Bakhtiari	NR	895263
[32]	Etemadifar M, 2014 (East Azerbaijan)	2006–2013	Population based	East Azerbaijan	NR	3724263
[32]	Etemadifar M, 2014 (Semnan)	2006–2013	Population based	Semnan	NR	631218
[32]	Etemadifar M, 2014 (Hamadan)	2006–2013	Population based	Hamadan	NR	1758268
[32]	Etemadifar M, 2014 (Qom)	2006–2013	Population based	Qom	NR	1151672
[32]	Etemadifar M, 2014 (West Azerbaijan)	2006–2013	Population based	West Azerbaijan	NR	3080576
[32]	Etemadifar M, 2014(Yazd)	2006–2013	Population based	Yazd	NR	1074428
[32]	Etemadifar M, 2014(Kordestan)	2006–2013	Population based	Kordestan	NR	1493645
[32]	Etemadifar M, 2014(Ardabil)	2006–2013	Population based	Ardabil	NR	1248488
[32]	Etemadifar M, 2014 (Kohgiluyeh)	2006–2013	Population based	Kohgiluyeh and Boyer-Ahmad	NR	658621
[32]	Etemadifar M, 2014 (Mazandaran)	2006–2013	Population based	Mazandaran	NR	3073943
[32]	Etemadifar M, 2014 (Guilan)	2006–2013	Population based	Guilan	NR	2480874
[32]	Etemadifar M, 2014 (Kerman)	2006–2013	Population based	Kerman	NR	2938988
[32]	Etemadifar M, 2014 (Khorasan-Razavi)	2006–2013	Population based	Razavi Khorasan	NR	5994402
[32]	Etemadifar M, 2014 (Bushehr)	2006–2013	Population based	Bushehr	NR	103949
[32]	Etemadifar M, 2014 (Ilam)	2006–2013	Population based	Ilam	NR	557599
[32]	Etemadifar M, 2014 (Khuzestan)	2006–2013	Population based	Khuzestan	NR	4531720
[32]	Etemadifar M, 2014 (Golestan)	2006–2013	Population based	Golestan	NR	1777014
[32]	Etemadifar M, 2014 (Lorestan)	2006–2013	Population based	Lorestan	NR	1754244
[32]	Etemadifar M, 2014 (Zanjan)	2006–2013	Population based	Zanjan	NR	1015734
[32]	Etemadifar M, 2014(Kermanshah)	2006–2013	Population based	Kermanshah	NR	1945227
[32]	Etemadifar M, 2014 (Hormozgan)	2006–2013	Population based	Hormozgan	NR	1578183
[32]	Etemadifar M, 2014 (North Khorasan)	2006–2013	Population based	North Khorasan	NR	867727
[32]	Etemadifar M, 2014 (South Khorasan)	2006–2013	Population based	South Khorasan	NR	662534
[32]	Etemadifar M, 2014(Qazvin)	2006–2013	Population based	Qazvin	NR	1201565
[32]	Etemadifar M, 2014 (Sistan and Baluchestan)	2006–2013	Population based	Sistan and Baluchestan	NR	2534327
[44]	Saadat SMS, 2013	2010	Cross-sectional	Guilan	McDonald	2480874
[42]	Raiesi R, 2014	1991–2011	Population based	Kohgiluyeh and Boyer-Ahmad	McDonald	895263
[60]	Izadi S, 2015	2011	Population based	Fars	NR	4596658
[5]	Nedjat S, 2006	2006	Population based	Tehran	NR	7803883
[71]	Yousefi B, 2017	2005–2009	Population based	East Azerbaijan	McDonald	3724620
[71]	Yousefi B, 2017	2010–2014	Population based	East Azerbaijan	McDonald	3909652
[64]	Mazdeh M, 2016	2015–2018	Population based	Hamadan	McDonald	
[70]	Tolou-Ghamari Z, 2015	2010–2014	Population based	Isfahan	McDonald	4879312
[69]	Shahbeigi S, 2012	2010–2014	Population based	12 different major provinces of Iran	McDonald	46695319
[63]	Khamarnia M, 2016	2010	Population based	Fars	McDonald	4596658
[63]	Khamarnia M, 2016	2011	Population based	Fars	McDonald	4596658
[63]	Khamarnia M, 2016	2012	Population based	Fars	McDonald	4596658
[13]	Dehghani R, 2015	2006	Population based	All Iran	McDonald	70495782
[13]	Dehghani R, 2015	2011	Population based	All Iran	McDonald	75149669
[57]	Eskandarieh Sh, 2017	2013	Population based	Tehran	McDonald	12559000
[57]	Eskandarieh Sh, 2017	2014	Population based	Tehran	McDonald	12559000
[57]	Eskandarieh Sh, 2017	2014	Population based	Tehran	McDonald	12559000
[66]	Sabbagh S, 2017	2015–2018	Population based	Khuzestan	McDonald	957133
[65]	Mousavizadeh A, 2017	2015–2018	Population based	Kohgiluyeh and Boyer-Ahmad	McDonald	713052
[56]	Eskandarieh Sh, 2017	2015	Population based	Tehran	McDonald	13267637
[58]	Eskandarieh Sh, 2018	2017	Population based	Tehran	McDonald	13441124

**NR:** Not reported

* Repetitive studies have been included and estimated the prevalence and incidence for more than 1 year and also regions. Each data was considered separately because of assessing the slope of prevalence and incidence in the years and estimating which region is the highest or lowest.

### 2.7. Data analysis

To evaluate the heterogeneity of the studies, Cochran's Q and I^2^ tests were used. Heterogeneity was defined as I^2^> 50% and the Cochran's Q test was defined as < 0.05. Therefore, the random effects model was used to estimate the prevalence and incidence of MS with high heterogeneity. To estimate the effect of gender, we used the total number and the number of events (MS) in men and women groups and we calculated the odds ratio (OR) and 95% CI. In this study, a sensitivity analysis was also performed to verify the stability of the data. In order to find the source of heterogeneity, a subgroup analysis was conducted in terms of geographic area, year of study, province, and study setting while a meta-regression model was used for the prevalence and incidence of MS in terms of year of studies. Begg and Egger’s tests were used to assess publication bias. Data were analyzed using Comprehensive meta-analysis ver. 2 software. P<0.05 was considered significant.

## 3. Results

### 3.1. Study characteristics and methodological quality

**Fig 1:**
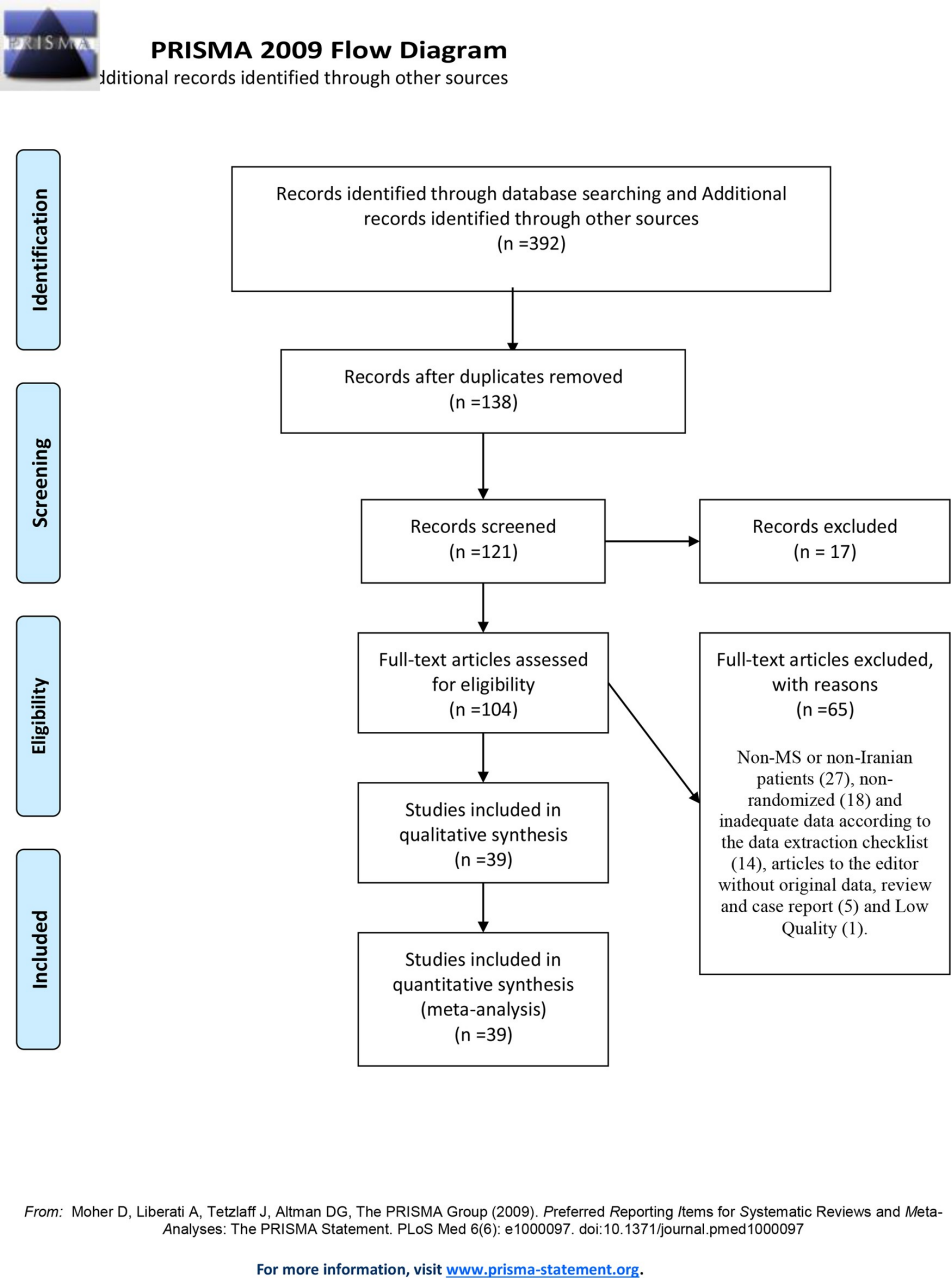
Study flow diagram.

Of 392 studies found in the initial search using the search strategy, 138 potentially relevant studies were found to be eligible for retrieval and evaluation. By examining the full text of the studies, 65 studies were excluded due to non-MS or non-Iranian patients (27), non-randomized (18) and inadequate data according to the data extraction checklist (14), articles to the editor without original data, review and case report (5) and low quality (1). Finally, 39 articles (included 103 studies for prevalence and 34 studies for incidence) entered the meta-analysis process after qualitative assessment. The flow diagram of the identification and selection of studies is illustrated in (Fig 1) and the characteristics of studies are shown in (Table 1) [2, 5, 13, 25, 26, 31–37, 39, 40, 42–46, 53–71].

### 3.2. Pooled prevalence of MS and sensitivity analysis

**Fig 2:**
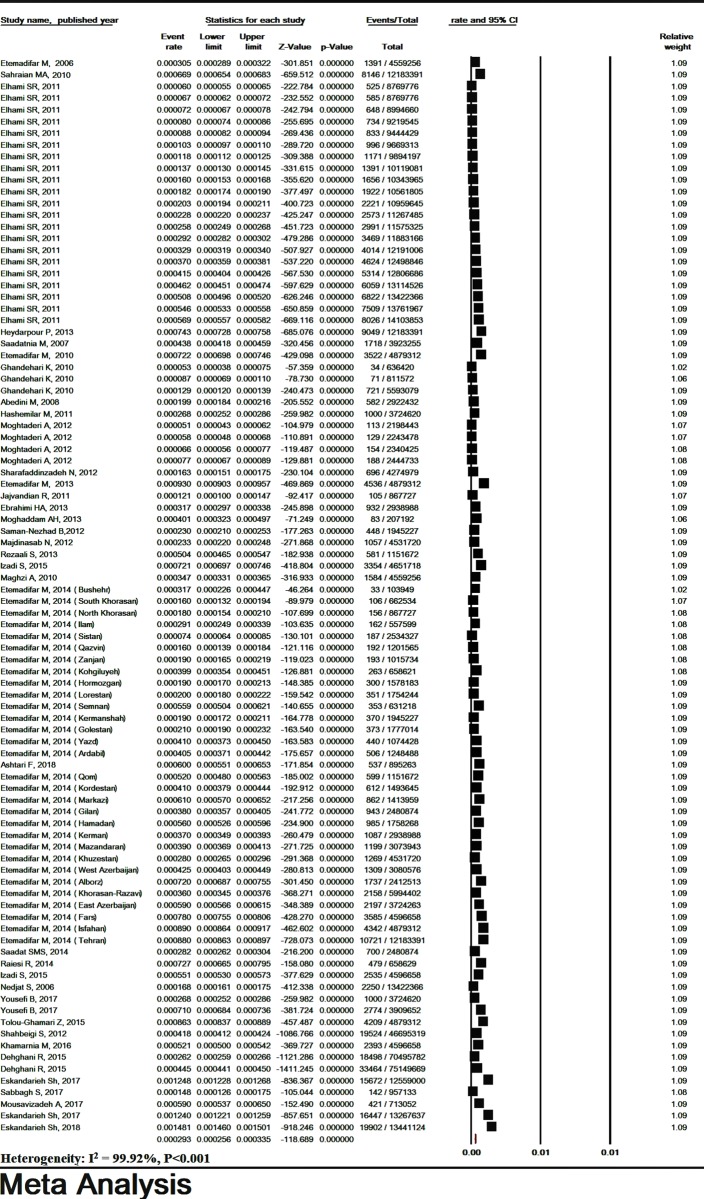
The prevalence of multiple sclerosis in Iran. Random effect model.

**Fig 3:**
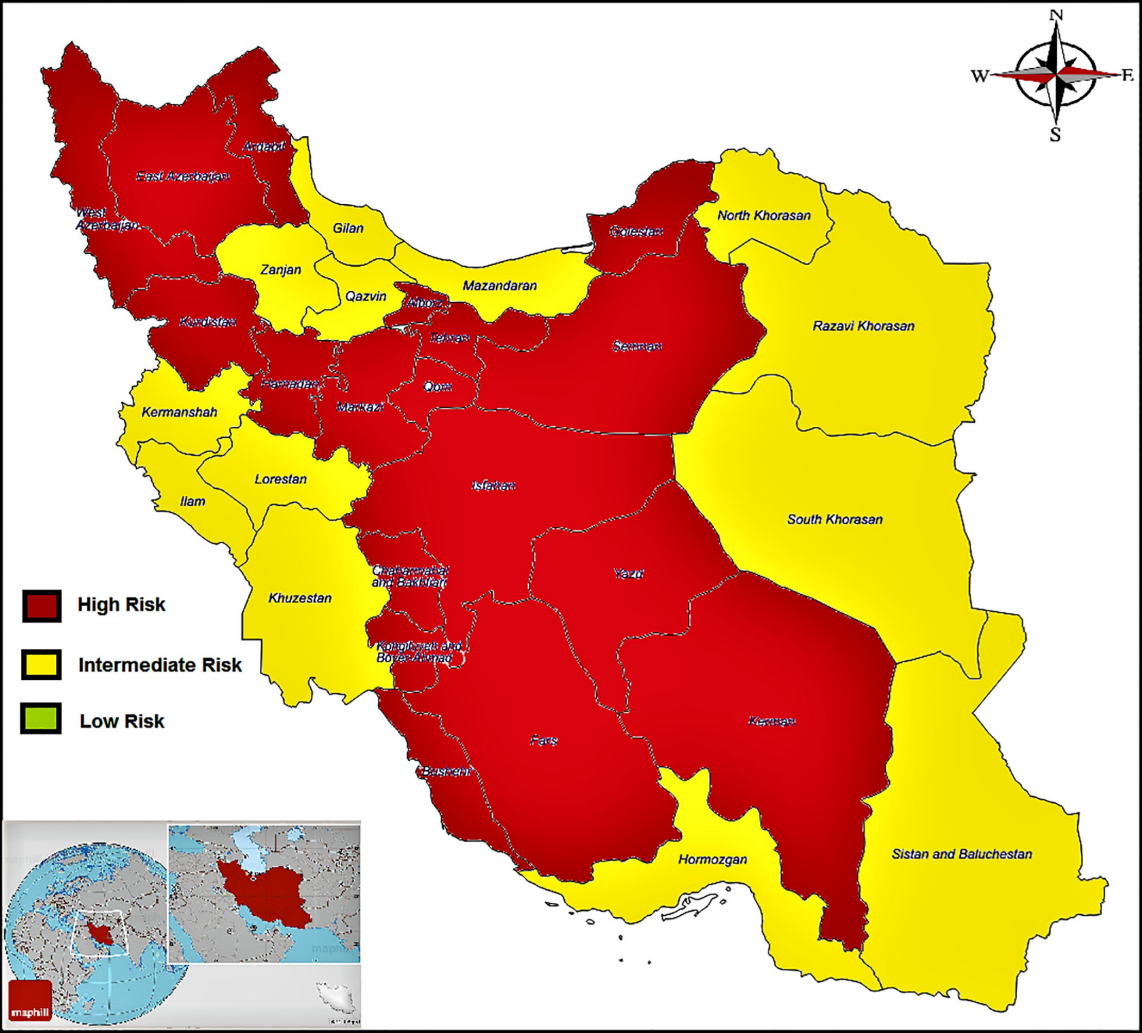
Distribution of MS in Iran based on geographical classification. High risk 30/100,000 intermediate risk 5-30/100,000 and low risk 5/100,000 (as Wade scaled prevalence of MS globally [72]).

The total heterogeneity was high (I^2^ = 99.92% and P< 0.001). The prevalence of MS in Iran was estimated to be 29.3/ 100,000 (95% CI: 25.6–33.5) based on random effects model (Fig 2). The lowest and highest prevalence was found in studies in Southern Khorasan in 2009 (5.3/ 100,000) and Isfahan in 2013 (89/ 100,000), respectively (Figs 2 and 3). The sensitivity analysis of the prevalence of MS and its 95% CI was estimated irrespective of one study at a time, and the results showed that the pooled estimate was robust (S1 Fig).

### 3.3. Subgroup analysis of MS prevalence based on region, province, study design, and year of study

The Subgroup analysis of MS prevalence in Iran is shown in (Table 2) and (S1–S6 Figs). Significant difference was observed in the prevalence of MS in the geographical regions (P< 0.001) (S2 Fig), province (P< 0.001) (S3 Fig), study design (P = 0.015) (S4 Fig), and year of study (P< 0.001) (S5 Fig).

**Table 2:** MS prevalence based on region, gender, provinces, year of study and design.

Variable		Studies (N)*	Sample (N)		Heterogeneity		95% CI	Pooled (Per 100,000)
			MS	All	I^2^	P-Value		
Region	12 different	1	19524	46695319	-	-	41.2–42.4	42.4
	All Iran	2	51962	145645451	99.97	< 0.001	20.4–57.4	34.2
	Center	47	174229	368149059	99.94	< 0.001	28.6–43.1	35.1
	East	8	3756	24011421	99.62	< 0.001	5.3–18.1	9.8
	North	14	11640	32250226	99.50	< 0.001	18.7–32.0	24.5
	South	13	17466	40504544	99.69	< 0.001	25.6–44.6	33.8
	West	7	4237	12534786	98.01	< 0.001	22.4–41.9	30.6
	Test for subgroup differences: Q = 45.66, df(Q) = 6, P< 0.001							
Gender	Male	31	25711	76907082	94.77	<0.0001	13.3–20.5	16.5
	Female	31	74356	75432131	99.87	< 0.001	36.2–55.4	44.8
	Rate ratio of female to male: OR = 3.01 (2.79–2 = 3.24, P<0.001)							
Province	12 different major provinces of Iran	1	19524	46695319	-	-	41.2–42.4	41.8
	Alborz	1	1737	2412513	-	-	68.7–75.5	72.0
	All Iran	2	51962	145645451	99.97	< 0.001	20.4–57.4	34.2
	Ardabil	1	506	1248488	-	-	37.1–44.2	40.5
	Bushehr	1	33	103949	-	-	22.6–44.7	31.7
	Chahar Mahaal and Bakhtiari	1	537	895263	-	-	55.1–65.3	60.0
	East Azerbaijan	4	6971	1508315	99.74	< 0.001	25.8–67.4	41.7
	Fars	4	11867	18441692	99.12	< 0.001	52.3–76.9	63.4
	Golestan	1	373	1777014	-	-	19.0–23.2	21.0
	Guilan	2	1643	4961748	97.19	< 0.001	24.5–43.9	32.8
	Hamadan	1	985	1758268	-	-	52.6–59.6	56.0
	Hormozgan	1	300	1578183	-	-	17.0–21.3	19.0
	Ilam	1	162	557599	-	-	24.9–33.9	29.1
	Isfahan	7	21302	32559015	99.79	< 0.001	43.5–79.3	58.7
	Kerman	3	2102	6085168	85.39	< 0.001	31.0–40.4	35.4
	Kermanshah	2	818	3890454	86.51	< 0.001	17.4–25.3	21.0
	Khuzestan	4	3164	14295552	98.11	< 0.001	15.3–26.2	20.1
	Kohgiluyeh and Boyer-Ahmad	4	1163	2030302	96.72	< 0.001	40.3–76.8	55.7
	Kordestan	1	612	1493645	-	-	37.9–44.4	41.0
	Lorestan	1	351	1754244	-	-	18.0–22.2	20.0
	Markazi	1	862	1413959	-	-	57.0–65.2	61.0
	Mazandaran	2	1781	5996375	99.43	< 0.001	14.4–53.9	27.9
	North Khorasan	3	332	2547026	92.70	< 0.001	8.3–18.8	12.5
	Qazvin	1	192	1201565	-	-	13.9–18.4	16.0
	Qom	2	1180	2303344	0	0.60	48.8–54.2	51.2
	Razavi Khorasan	2	2879	11587481	99.82	< 0.001	7.9–59.0	21.6
	Semnan	1	353	631218	-	-	50.4–62.1	55.9
	Sistan and Balouchestan	5	771	11761406	75.34	< 0.001	5.6–7.5	6.5
	South Khorasan	2	140	1298954	99.77	< 0.001	3.2–27.3	9.3
	Tehran	28	146270	322611718	99.96	< 0.001	20.7–36.7	27.6
	West Azerbaijan	1	1309	3080576	-	-	20.7–36.7	27.6
	Yazd	1	440	1074428	-	-	37.3–45.0	41.0
	Zanjan	1	193	1015734	-	-	16.5–21.9	19.0
	Test for subgroup differences: Q = 2559.92, df(Q) = 32, P< 0.001							
Year of study	< 1990	2	1110	17539552	69.13	0.072	5.7–7.0	6.3
	1990–1994	5	4382	47222144	97.14	< 0.001	7.6–10.8	9.1
	1995–1999	5	9763	53251981	98.62	< 0.001	15.1–21.3	17.9
	2000–2004	5	20412	60955029	99.27	< 0.001	27.9–38.6	32.8
	2005–2009	20	58545	182277428	99.82	< 0.001	15.5–23.6	19.1
	2010–2014	51	151690	280165726	99.81	< 0.001	35.8–45.8	40.5
	2015–2018	4	36912	28378946	99.76	< 0.001	50.7–85.0	65.6
Test for subgroup differences: Q = 744.07, df(Q) = 6, P< 0.001							
Study design	Population based	82	266175	627821102	99.83	< 0.001	27.0–35.9	31.1
	Cross-sectional	10	16639	41969704	99.92	< 0.001	11.7–27.3	17.9
	Test for subgroup differences: Q = 5.89, df(Q) = 1, P = 0.015							

N: Number; CI: confidence interval

* Some studies have been included and estimated the prevalence and incidence for more than 1 year and also regions. Each data was considered separately because of assessing the slope of prevalence and incidence in the years and estimating which region is the highest or lowest.

### 3.4. Prevalence of MS based on gender

**Fig 4:**
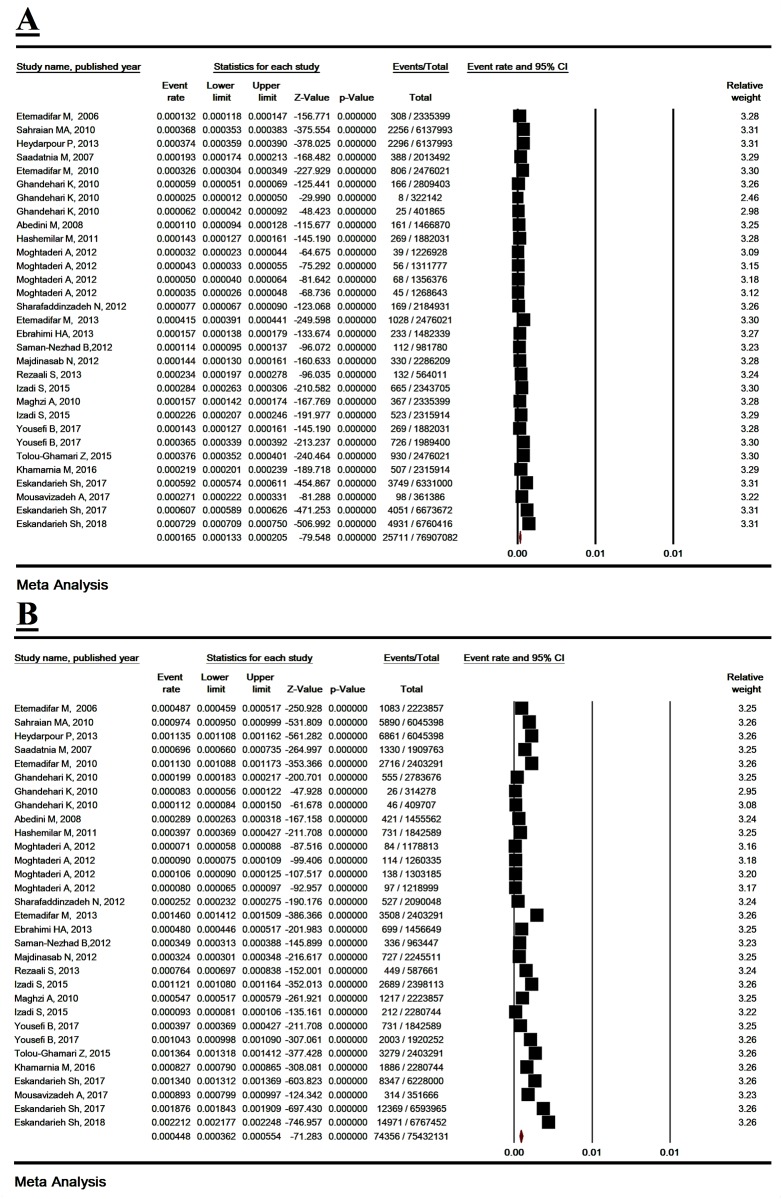
The prevalence of multiple sclerosis in men (A) and women (B). Random effect model.

The prevalence of MS in men and women was estimated to be 16.5/ 100,000 (95% CI: 13.7–23.4) and 44.8/ 100,000 (95% CI: 36.3–61.6), respectively (Fig 4). The OR female/ male of MS prevalence was estimated to be 3.01 (95% CI: 2.79–3.24, P< 0.001) (Table 2) (S6 Fig).

### 3.5. Pooled incidence of MS and sensitivity analysis

**Fig 5:**
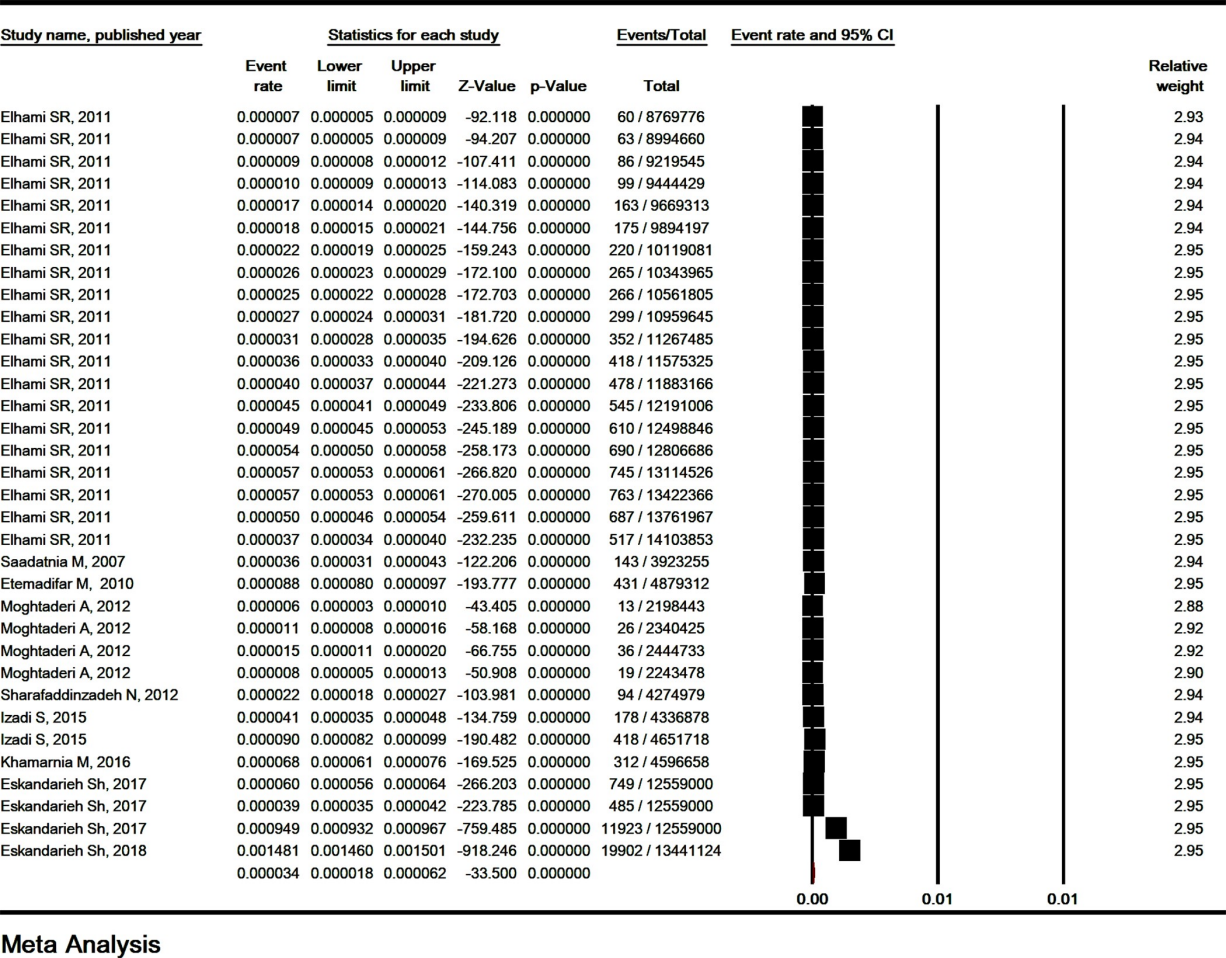
The incidence of multiple sclerosis in Iran. Random effect model.

The total heterogeneity was high (I^2^ = 99.96% and P< 0.001). The incidence of MS in Iran was estimated according to 34 studies to be 3.4/ 100,000 (95% CI: 1.8–6.2) based on random effects model (Fig 5). The sensitivity analysis results are shown in (S7 Fig).

### 3.6. Subgroup analysis of MS incidence based on region, province, study design, and year of study

The Subgroup analysis of MS incidence in Iran is shown in (Table 3). Significant difference was observed in the prevalence of MS in the geographical regions (P < 0.001) (S8 Fig), province (P< 0.001) (S9 Fig) and year of study (P< 0.001) (S10 Fig), but study design was no significant difference (P = 0.123) (S11 Fig).

**Table 3:** MS incidence based on region, gender, provinces, year of study and design.

Variable		Studies (N)*	Sample (N)		Heterogeneity		95% CI	Pooled (Per 100,000)
			MS	All	I^2^	P-Value		
Region	Center	26	41134	284522333	99.97	< 0.001	2.0–7.6	3.9
	East	4	94	9227079	67.80	0.025	0.7–1.4	1.0
	South	4	1002	17860233	98.43	< 0.001	2.9–8.2	4.9
	Test for subgroup differences: Q = 28.94, df(Q) = 2, P< 0.001							
Gender	Male	31	2158	136555427	99.87	< 0.001	1.0–1.4	1.2
	Female	31	76156	75432131	99.87	< 0.001	39.3–59.1	48.2
	Rate ratio of female to male: OR = 3.04 (2.85–3.24, P< 0.001)							
Province	Fars	3	908	13585254	97.40	< 0.001	4.2–9.6	6.3
	Isfahan	2	574	8802567	98.81	< 0.001	2.4–13.5	5.7
	Khuzestan	1	94	4274979	-	-	1.8–2.7	2.2
	Sistan and Balouchestan	4	94	9227079	67.80	0.025	0.7–1.4	1.0
	Tehran	24	40560	275719766	99.97	< 0.001	1.8–7.6	3.7
	Test for subgroup differences: Q = 49.07, df(Q) = 4, P< 0.001							
Year of study	< 1990	1	60	8769776	-	-	0.5–0.9	0.7
	1990–1994	5	586	47222144	93.90	< 0.001	0.8–1.6	1.2
	1995–1999	5	1402	53251981	79.37	< 0.001	2.3–2.9	2.6
	2000–2004	6	2919	65291907	90.76	< 0.001	3.9–5.0	4.4
	2005–2009	11	3474	76707337	98.09	< 0.001	2.2–3.7	2.8
	2010–2014	5	13887	46925376	99.96	< 0.001	2.0–56.7	10.6
	2015–2018	1	19902	13441124	-	-	146.0–150.1	148.1
	Test for subgroup differences: Q = 10943.73, df(Q) = 6, P< 0.001							
Study design	Population based	27	41468	289305020	98.66	< 0.001	2.0–7.7	4.0
	Cross-sectional	7	762	22304625	99.97	< 0.001	0.9–3.8	1.8
	Test for subgroup differences: Q = 2.38, df(Q) = 1, P = 0.123							

N: Number; CI: confidence interval

* Some studies have been included and estimated the prevalence for more than 1 year and also regions. Each data was considered separately because of assessing the slope of prevalence in the years and estimating which region is the highest or lowest.

### 3.7. Incidence of MS based on gender

**Fig 6:**
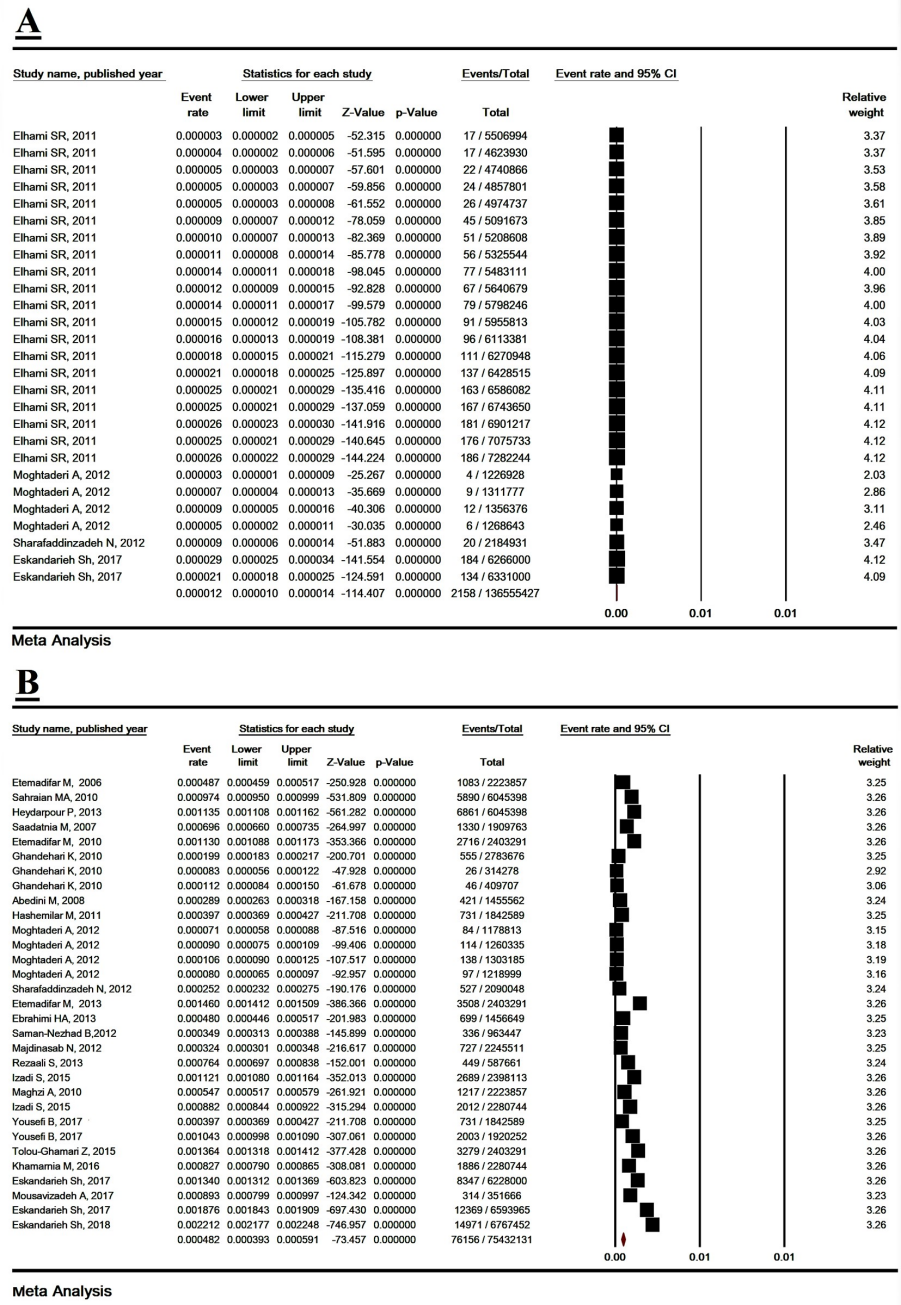
The incidence of multiple sclerosis in men (A) and women (B). Random effect model.

The incidence of MS in men was estimated to be 16.5/ 100,000 (95% CI: 13.7–23.4) and the incidence of MS in women was 44.8/ 100,000 (95% CI: 36.3–61.6) (Fig 6). The OR female/male of MS incidence was estimated to be 3.04 (2.85–3.24, P< 0.001) (Table 2) (S12 Fig).

### 3.8. Meta-regression

**Fig 7:**
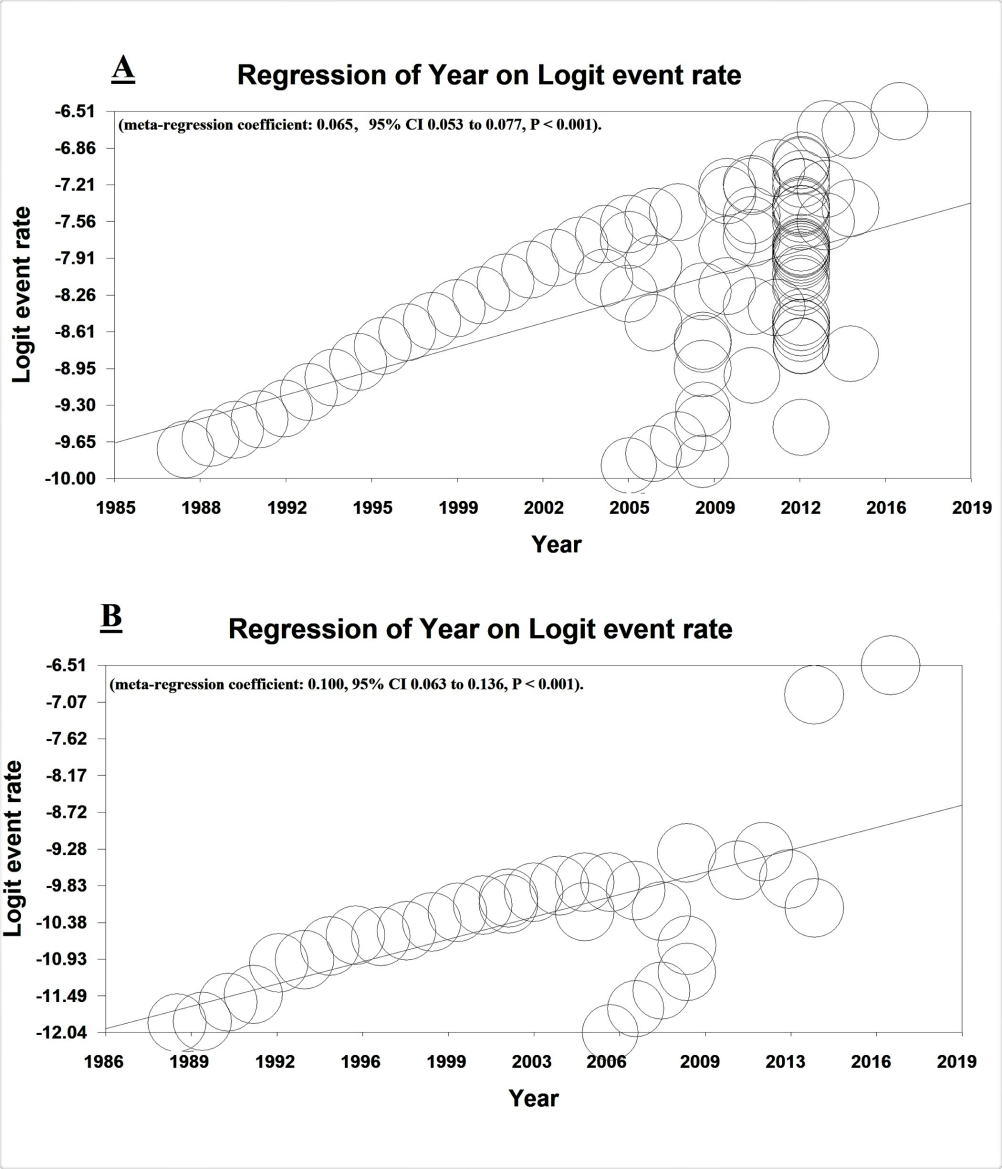
Meta-regression of MS in Iran according to year of studies. Prevalence (A) and incidence (B).

The meta-regression model for prevalence and incidence of MS was significantly higher in terms of year of study [(meta-regression coefficient: 0.065, 95% CI 0.053 to 0.077, P< 0.001) for prevalence of MS and (meta-regression coefficient: 0.100, 95% CI 0.063 to 0.136, P< 0.001) for incidence of MS] (Fig 7). Moreover, the meta-regression model for prevalence and incidence of MS based on the year was also studied in men [(meta-regression coefficient: 0.202, 95% CI 0.157 to 0.248, P< 0.001) for prevalence of MS and (meta-regression coefficient: 0.065, 95% CI 0.046 to 0.0840, P< 0.001) for incidence of MS] and women [(meta-regression coefficient: 0.216, 95% CI 0.169 to 0.264, P< 0.001) for prevalence of MS and (meta-regression coefficient: 0.219, 95% CI 0.176 to 0.263, P< 0.001) for incidence of MS] and it was increasing significantly (S13 and S14 Figs).

### 3.9. Publication bias

Publication bias in the studies of incidence (Egger< 0.001, and Begg’s< 0.001) and prevalence (Egger< 0.001, and Begg’s = 0.045) of MS was significant (S15 Fig).

## 4. Discussion

The present study is the first systematic review and meta-analysis on the epidemiology of MS in Iran. According to the results of the present meta-analysis, the prevalence and incidence of MS in Iran is estimated to be 29.3/ 100,000 and 3.4/ 100,000, which is more than some Middle Eastern countries (Oman, Libya, Lebanon, Iraq, Kuwait, and Tunisia)[73–78] and less than some other countries (UAE, city of Amman in Jordan and Saudi Arabia) [79–81]. However, it should be noted that most studies in our meta-analysis process were based on data from MS centers, and the lack of recording the information of some people with MS was due to non-compulsory membership in this center and the real prevalence of MS is expected to be greater than this figure. In 2016, Nasr et al. [41] investigated the prevalence of MS among Iranian migrants. The prevalence of MS among Iranian migrants was 21/ 100,000 in Mumbai (India) in 1985 and 433/ 100,000 in British Columbia (Canada) in 2012. In five different studies, the MS prevalence in the studied areas was reported from 1.33 in Mumbai (India) to 240 in British Columbia (Canada)[82–86]. The acculturative stress in migrants may help to relate the onset of illness and migration. The acculturative stress is the tension or pressure associated with the experience of a second culture that may have adverse effects on physical or mental health [87] and shows that stress and anxiety have a potential role in MS development[88]. Nish et al. recently showed in a study that acculturative stress is related to higher inflammatory markers in a Chinese migrant population[89].

In the past, the behavior and distribution of MS disease were associated with latitude and was reported to be lower in areas with a higher latitude. Overall, according to a report by WHO in 2008, the highest reported MS prevalence was in North America and Europe, and the lowest reported MS prevalence was in countries near the equator. However, this pattern is changing and areas with lower prevalence are changing to areas with higher prevalence[10, 15, 18, 90]. In the present study, there was a significant difference between the five geographical regions of Iran in terms of the prevalence and incidence of MS based on the results of the initial studies.

Based on the present meta-analysis, the OR of prevalence and incidence of MS in women was 2.52 and 3.04, respectively compared with men, which was a significant relationship (P< 0.001). This result is similar to the results in previous studies [21, 38, 55, 91–93].

According to the meta-regression model, the prevalence and incidence of MS in Iran increased significantly (p< 0.001) with an increase in year of studies[21, 38, 91–93].

Various factors such as the lack of prevention and screening programs can be important factors in increasing the prevalence of the disease. In addition to changes in the pattern of food consumption, food quality has also changed a lot recently [10, 13, 30]. According to the WHO, the use of tobacco, fat, salt and sugar higher than the limit in foods that cause overweight and obesity, industrialization, urbanization and economic development can play a significant role in the development of chronic diseases[94]. In a study in the United States on 8983 MS patients, it was found that 25% of patients were obese and 31.3% were overweight. In addition, 18.2% were at risk of alcohol misuse by themselves or their relatives[95].

Since there are no particular laws and regulations on the purchase and use of chemicals in Iran, they are easily accessible to people and this may increase the risk of diseases such as MS, which is caused by exposure to chemicals. Although in some studies, contact with industrial solvents has been identified as a risk factor for MS, it has not yet been confirmed for sure[96–98].

The existence of particles such as PM10 in the air of Iranian cities[10, 28, 30, 99–102], natural radiation of radon from soil (Ramsar, Iran) [103] and unsupervised use of decorative stones and granite in Iran[27, 104] may increase the risk of MS. However, few studies have been conducted regarding the relationship between the above parameters and MS.

According to the Ministry of Health in Iran, the rate of smoking has risen to about 60 billion cigarettes per year[29, 105]. Inhaling cigarette smoke exacerbates the effect on chronic diseases [106, 107]. According to an ecological study by Dehghani et al. in Iran, the prevalence of illness is higher in provinces where cigarette smoking is higher among males[13]. Since cigarette smoking increases the frequency and duration of respiratory infections and it causes MS relapse[37], the risk of cigarette smoking for MS with an OR of 1.55, 95% CI [1.48–1.62], P<0.001 was confirmed in the recent meta-analysis.

According to the WHO, the prevalence of MS is higher in countries with higher income levels. However, the diseases may progress more in less developed countries due to less access to diagnostic facilities, although the disparity is so high that scarce diagnostic facilities cannot be considered as the main factor[108].

Studies have shown that vitamin D deficiency is inversely related to the risk of MS [109, 110] and its deficiency is an epidemic, which affects 20–25% of the population in Asia, America, Canada, Europe and Australia[111]. This is becoming acuter in the Middle East because of changes in lifestyle conditions and less sunlight[112]. Systematic reviews and meta-analyses in Iran have reported a high prevalence of vitamin D deficiency[91, 92].

The period of MS is often unpredictable, but some factors can predict a patient's prognosis. The indicators of a good prognosis can be female gender, those with a history of disease before the age of 35, those who were only attacked in one area of the brain, those who had no brain stem involvement and patients who had recovered after the attacks[10]. To achieve successful symptom control, multiple controls are needed to prevent or stop the symptoms. Effective communication, training, exercise, professional support, and pharmacological interventions are vital for effective control of multiple sclerosis symptoms.

## 5. Limitations

1. The insensitivity of internal databases to operators “AND” and “OR” to search for the combination. 2. Since Tehran is the main medical center of many cities and provinces, patients in the studies in Tehran, are not just from Tehran.

3. No separation of rural and urban prevalence of MS

## 6. Conclusion

The present meta-analysis showed that the prevalence and incidence of MS in Iran is high (as Wade scaled prevalence of MS globally[72]) and is rising over time. The results of this study provide useful information for neurologists and health policy makers and can provide a general overview of MS epidemiology in Iran.

## Appendix 1: PubMed search strategy

1. Exp. 'Epidemiology'
2. Exp.'Prevalence'
3. Exp.'Incidence’'
4. Exp.MS'
5. Exp.'Multiple Sclerosis '
6. Exp.'Iran'
7. 1 OR 2 OR 3
8. 4 OR 5
9. 7 AND 8 AND 9

## Supporting information

S1 File PRISMA checklist. (DOC)

S2 File The review protocol which has been registered in PROSPERO International Prospective Register of Systematic Reviews. (PDF)

S3 File Newcastle-Ottawa scale checklist. (PDF)

S1 Fig Sensitivity analysis for prevalence of multiple sclerosis in Iran. (TIF)

S2 Fig Subgroup analysis for multiple sclerosis prevalence based on region. (TIF)

S3 Fig Subgroup analysis for multiple sclerosis prevalence based on province. (TIF)

S4 Fig Subgroup analysis for multiple sclerosis prevalence based on study design. (TIF)

S5 Fig Subgroup analysis for multiple sclerosis prevalence based on study year. (TIF)

S6 Fig The OR female to male of MS prevalence (A) and incidence (B).
(TIF)

S7 Fig Sensitivity analysis for prevalence of multiple sclerosis in Iran. (TIF)

S8 Fig Subgroup analysis for multiple sclerosis incidence based on region. (TIF)

S9 Fig Subgroup analysis for multiple sclerosis incidence based on province. (TIF)

S10 Fig Subgroup analysis for multiple sclerosis incidence based on study year. (TIF)

S11 Fig Subgroup analysis for multiple sclerosis incidence based on study design. (TIF)

S12 Fig The OR female/male of MS incidence. (TIF)

S13 Fig Prevalence of Multiple Sclerosis in Iran in terms of men (A) and women (B).
(TIF)

S14 Fig Incidence of Multiple Sclerosis in Iran in terms of men (A) and women (B).
(TIF)

S15 Fig Publication bias for prevalence studies (A) and update (B) multiple sclerosis in Iran.
(TIF)
