# Correction: Epidemiology of multiple sclerosis in Iran: A systematic review and meta-analysis

**DOI:** 10.1371/journal.pone.0219466

**Published:** 2019-07-09

**Authors:** Milad Azami, Mohammad Hossein YektaKooshali, Masoumeh Shohani, Ali Khorshidi, Leily Mahmudi

In the Results subsection of the Abstract, there are errors in the third and fourth sentence of the paragraph. The correct sentences are: The prevalence of MS in men and women was estimated to be 16.5/ 100,000 (95% CI: 13.3–20.5) and 44.8/ 100,000 (95% CI: 36.2–55.4), respectively. The incidence of MS in men was estimated to be 1.2/ 100,000 (95% CI: 1.0–1.4) and the incidence of MS in women was 48.2/ 100,000 (95% CI: 39.3–59.1).

In the 3.1. Study characteristics and methodological quality subsection of the Results, there is an error in the third sentence of the paragraph. The correct sentence is: Finally, 39 articles (included 92 studies for prevalence and 34 studies for incidence) entered the meta-analysis process after qualitative assessment.

In the 3.4. Prevalence of MS based on gender subsection of the Results, there is an error in the first sentence of the paragraph. The correct sentence is: The prevalence of MS in men and women was estimated to be 16.5/ 100,000 (95% CI: 13.3–20.5) and 44.8/ 100,000 (95% CI: 36.2–55.4), respectively (Fig 4).

In the 3.7. Incidence of MS based on gender subsection of the Results, there is an error in the first sentence of the paragraph. The correct sentence is: The incidence of MS in men was estimated to be 1.2/ 100,000 (95% CI: 1.0–1.4) and the incidence of MS in women was 48.2/ 100,000 (95% CI: 39.3–59.1) (Fig 6).

There is an error in Table 2. Please see the correct Table 2 here.

**Table 2:** MS prevalence based on region, gender, provinces, year of study and design.

Variable		Studies (N)*	Sample (N)		Heterogeneity		95% CI	Pooled (Per 100,000)
			MS	All	I^2^	P-Value		
Region	12 different	1	19524	46695319	-	-	41.2–42.4	42.4
	All Iran	2	51962	145645451	99.97	< 0.001	20.4–57.4	34.2
	Center	47	174229	368149059	99.94	< 0.001	28.6–43.1	35.1
	East	8	3756	24011421	99.62	< 0.001	5.3–18.1	9.8
	North	14	11640	32250226	99.50	< 0.001	18.7–32.0	24.5
	South	13	17466	40504544	99.69	< 0.001	25.6–44.6	33.8
	West	7	4237	12534786	98.01	< 0.001	22.4–41.9	30.6
	Test for subgroup differences: Q = 45.66, df(Q) = 6, P< 0.001							
Gender	Male	31	25711	76907082	94.77	<0.0001	13.3–20.5	16.5
	Female	31	74356	75432131	99.87	< 0.001	36.2–55.4	44.8
	Rate ratio of female to male: OR = 3.01 (2.79–2 = 3.24, P<0.001)							
Province	12 different major provinces of Iran	1	19524	46695319	-	-	41.2–42.4	41.8
	Alborz	1	1737	2412513	-	-	68.7–75.5	72.0
	All Iran	2	51962	145645451	99.97	< 0.001	20.4–57.4	34.2
	Ardabil	1	506	1248488	-	-	37.1–44.2	40.5
	Bushehr	1	33	103949	-	-	22.6–44.7	31.7
	Chahar Mahaal and Bakhtiari	1	537	895263	-	-	55.1–65.3	60.0
	East Azerbaijan	4	6971	1508315	99.74	< 0.001	25.8–67.4	41.7
	Fars	4	11867	18441692	99.12	< 0.001	52.3–76.9	63.4
	Golestan	1	373	1777014	-	-	19.0–23.2	21.0
	Guilan	2	1643	4961748	97.19	< 0.001	24.5–43.9	32.8
	Hamadan	1	985	1758268	-	-	52.6–59.6	56.0
	Hormozgan	1	300	1578183	-	-	17.0–21.3	19.0
	Ilam	1	162	557599	-	-	24.9–33.9	29.1
	Isfahan	7	21302	32559015	99.79	< 0.001	43.5–79.3	58.7
	Kerman	3	2102	6085168	85.39	< 0.001	31.0–40.4	35.4
	Kermanshah	2	818	3890454	86.51	< 0.001	17.4–25.3	21.0
	Khuzestan	4	3164	14295552	98.11	< 0.001	15.3–26.2	20.1
	Kohgiluyeh and Boyer-Ahmad	3	1163	2030302	96.72	< 0.001	40.3–76.8	55.7
	Kordestan	1	612	1493645	-	-	37.9–44.4	41.0
	Lorestan	1	351	1754244	-	-	18.0–22.2	20.0
	Markazi	1	862	1413959	-	-	57.0–65.2	61.0
	Mazandaran	2	1781	5996375	99.43	< 0.001	14.4–53.9	27.9
	North Khorasan	3	332	2547026	92.70	< 0.001	8.3–18.8	12.5
	Qazvin	1	192	1201565	-	-	13.9–18.4	16.0
	Qom	2	1180	2303344	0	0.60	4.03–44.9	42.5
	Razavi Khorasan	2	2879	11587481	99.82	< 0.001	7.9–59.0	21.6
	Semnan	1	353	631218	-	-	50.4–62.1	55.9
	Sistan and Balouchestan	5	771	11761406	75.34	< 0.001	5.6–7.5	6.5
	South Khorasan	2	140	1298954	99.77	< 0.001	3.2–27.3	9.3
	Tehran	28	146270	322611718	99.96	< 0.001	20.7–36.7	27.6
	West Azerbaijan	1	1309	3080576	-	-	4.03–44.9	42.5
	Yazd	1	440	1074428	-	-	37.3–45.0	41.0
	Zanjan	1	193	1015734	-	-	16.5–21.9	19.0
	Test for subgroup differences: Q = 2559.92, df(Q) = 32, P< 0.001							
Year of study	< 1990	2	1110	17539552	69.13	0.072	5.7–7.0	6.3
	1990–1994	5	4382	47222144	97.14	< 0.001	7.6–10.8	9.1
	1995–1999	5	9763	53251981	98.62	< 0.001	15.1–21.3	17.9
	2000–2004	5	20412	60955029	99.27	< 0.001	27.9–38.6	32.8
	2005–2009	20	58545	182277428	99.82	< 0.001	15.5–23.6	19.1
	2010–2014	51	151690	280165726	99.81	< 0.001	35.8–45.8	40.5
	2015–2018	4	36912	28378946	99.76	< 0.001	50.7–85.0	65.6
Test for subgroup differences: Q = 744.07, df(Q) = 6, P< 0.001								
Study design	Population based	82	266175	627821102	99.83	< 0.001	27.0–35.9	31.1
	Cross-sectional	10	16639	41969704	99.92	< 0.001	11.7–27.3	17.9
	Test for subgroup differences: Q = 5.89, df(Q) = 1, P = 0.015							

N: Number; CI: confidence interval

* Some studies have been included and estimated the prevalence and incidence for more than 1 year and also regions. Each data was considered separately because of assessing the slope of prevalence and incidence in the years and estimating which region is the highest or lowest

## References

[1] Azami M, YektaKooshali MH, Shohani M, Khorshidi A, Mahmudi L (2019) Epidemiology of multiple sclerosis in Iran: A systematic review and meta-analysis. PLoS ONE 14(4): e0214738 10.1371/journal.pone.0214738 30964886 PMC6456231

