# Supplementary material for: Epidemiology of multiple sclerosis in Iran: A systematic review and meta-analysis
Source: PLoS One. 2019 Apr 9;14(4):e0214738. doi: 10.1371/journal.pone.0214738 (PMC6456231; doi:10.1371/journal.pone.0214738)
Supplement: S2 File — (PDF) [file pone.0214738.s002.pdf]

Epidemiology of multiple sclerosis in Iran: a systematic review and ?meta-analysis

Leliy Mahmmudi, Masoumeh Shohani, Mohammad Hossein YektaKooshali, Milad Azami

Citation

Leliy Mahmmudi, Masoumeh Shohani, Mohammad Hossein YektaKooshali, Milad Azami. Epidemiology of multiple sclerosis in Iran: a systematic review and ?meta-analysis. PROSPERO 2018 CRD42018114491 Available from:
http://www.crd.york.ac.uk/PROSPERO/display_record.php?ID=CRD42018114491

Review question

What is the overall prevalence of MS and its sensitivity analysis?

What is the prevalence of MS based on region, ?province, study design, and year of study?

What is the prevalence of MS based on gender?

What is the overall incidence of MS and its sensitivity analysis?

What is the incidence of MS based on region, province, ?study design, and year of study?

What is the Incidence of MS based on gender?

?What is the the meta-regression diagram for prevalence and incidence of MS based on the year??

?How is the the publication bias in the studies of incidence and prevalence?

what is the result of GRADE assessment of confidence in estimates of effect ?(Grading of evidence)?

Searches

The present systematic review focused on MS epidemiology in Iran based on PRISMA guideline ??for systematic review and meta-analysis. All the steps of research, including search, ?selection of studies, qualitative assessment, and data extraction will be carried out independently by two ?researchers. ?

The search was performed by two researchers independently. We searched the titles and abstracts ?of articles in six Persian databases including Scientific Information Database (SID) ??(http://www.sid.ir/), Barakat Knowledge Network System (http://health.barakatkns.com), ??(Iranian Research Institute for Information Science and Technology (IranDoc) ??(https://irandoc.ac.ir), Regional Information Center for Science and Technology (RICST) ??(http://en.ricest.ac.ir/), Magiran (http://www.magiran.com/), Iranian National Library ??(http://www.nlai.ir/) and seven international databases including Scopus, PubMed/MEDLINE, ?ScienceDirect, Cochrane Library, Web of Science, Embase, PsycINFO as well as Google ?Scholar search engine for peer-reviewed studies published without time limit until May 2018.

The ?keywords used were 'incidence', 'prevalence', 'epidemiology', 'MS', 'multiple sclerosis' and 'Iran'. ?Boolean operators (AND & OR) were used to search by a combination of words.

Types of study to be included

Cross-sectional and epidemiological studies

Condition or domain being studied

Epidemiology of multiple sclerosis in Iran containing prevalence and incidence.

Participants/population

All epidemiological studies on MS? in Iran.

Intervention(s), exposure(s)
Intervention: Poser or McDonald criteria for confirmed MS

Comparator(s)/control
That can show the prevalence and incidence of MS based on geographical region and province ?and other mentioned subgroups.

Context

Main outcome(s)
Overall prevalence and incidence of MS in Iran

Additional outcome(s)
Overall prevalence of MS and its sensitivity analysis.

Prevalence of MS based on region, ?province, study design, and year of study.

Prevalence of MS based on gender.

Overall incidence of MS and its sensitivity analysis.

Incidence of MS based on region, province, ?study design, and year of study.

Incidence of MS based on gender.

The meta-regression diagram for prevalence and incidence of MS based on the year.

Publication bias in the studies of incidence and prevalence.

Result of GRADE assessment of confidence in estimates of effect ?(Grading of evidence).

Data extraction (selection and coding)
The following information will be extracted:

First author, year of publication, year of study, study setting, location, sample size, geographical ?area, province, the prevalence and incidence of MS and MS diagnostic method.

Risk of bias (quality) assessment
Researchers will be examining the quality of the selected articles using a scoring system based on the 8-?item the modified Newcastle Ottawa Scale (NOS) for non-randomized studies. ?Each question will be given a score between 0 and 1. Points 0-5, 6-7 and 8-9 will be considered low ?quality, moderate quality and high quality, respectively. The minimum score for entering the ?quantitative meta-analysis process will be 5 and the articles that will be acquired the minimum qualitative ?assessment score entered the process of data extraction and meta-analysis. ?

Strategy for data synthesis
The present systematic review focused on MS epidemiology in Iran based on PRISMA guideline ??for systematic review and meta-analysis. All the steps of research, including search, ?selection of studies, qualitative assessment, and data extraction will be carried out independently by two ?researchers, in the event of any differences in opinion, a specialist will carefully investigate the issue.

We will provide a narrative synthesis of the results from the included studies.

We searched the titles and abstracts ?of articles in six Persian databases and seven international databases for peer-reviewed studies published without time limit until May 2018. 'incidence', 'prevalence', 'epidemiology', 'MS', 'multiple sclerosis' and 'Iran'. ?Boolean operators (AND & OR) used to search by a

combination of words.

To evaluate the heterogeneity of the studies, Cochran's Q and I^2 tests will be use. Heterogeneity ?will be defined as $I^2>50\%$ and the Cochran's Q test was defined as <0.05. Therefore, the random ?effects model will be use to estimate the prevalence of MS with high heterogeneity. In this study, ?a sensitivity analysis will be also perform to verify the stability of the data. In order to find the ?source of heterogeneity, a subgroup analysis will be conduct in terms of geographic area, year of ?study, province, and study setting while a meta-regression model will be use for the prevalence of ?MS in terms of year of studies. Begg and Egger’s tests will be use to assess publication bias. Data ?will be analyse using comprehensive meta-analysis version 2 software. $P<0.05$ will be considered ?significant.?

Analysis of subgroups or subsets

The following subgroup analyses will be carried out:

Overall prevalence of MS and its sensitivity analysis;

Prevalence of MS based on region, ?province, study design, and year of study;

Prevalence of MS based on gender;

Overall incidence of MS and its sensitivity analysis;

Incidence of MS based on region, province, ?study design, and year of study;

Incidence of MS based on gender;

The meta-regression diagram for prevalence and incidence of MS based on the year;

Publication bias in the studies of incidence and prevalence;

Result of GRADE assessment of confidence in estimates of effect ?(Grading of evidence);

Contact details for further information

Mohammad Hossein YektaKooshali
Yektakooshali.mh1995@yahoo.co.uk

Organisational affiliation of the review

Guilan University of Medical Sciences, Rasht, Iran

Review team members and their organisational affiliations

Dr Leliy Mahmmudi. Faculty of Medicine, Dezful University of Medical Sciences, Dezful, Iran.
Dr Masoumeh Shohani. Department of Nursing, Faculty of Allied Medical Sciences, Ilam University of Medical Sciences, Ilam, Iran.
Dr Mohammad Hossein YektaKooshali. Guilan University of Medical Sciences, Rasht, Iran
Dr Milad Azami. School of Medicine, Ilam University of Medical Sciences, Ilam, Iran.

Anticipated or actual start date

17 April 2018

Anticipated completion date

22 November 2018

Funding sources/sponsors

None

Conflicts of interest

None known.

Language
English

Country
Iran

Stage of review
Review_Ongoing

Subject index terms status
Subject indexing assigned by CRD

Subject index terms
Humans; Iran; Multiple Sclerosis; Prevalence

Date of registration in PROSPERO
08 November 2018

Date of publication of this version
04 December 2018

Details of any existing review of the same topic by the same authors

Stage of review at time of this submission

Stage	Started	Completed
Preliminary searches	Yes	Yes
Piloting of the study selection process	Yes	No
Formal screening of search results against eligibility criteria	Yes	No
Data extraction	No	No
Risk of bias (quality) assessment	No	No
Data analysis	No	No

Versions
08 November 2018
04 December 2018

PROSPERO
This information has been provided by the named contact for this review. CRD has accepted this information in good faith and registered the review in PROSPERO. CRD bears no responsibility or liability for the content of this registration record, any associated files or external websites.
