# Supplementary figures and images for: Epidemiology of multiple sclerosis in Iran: A systematic review and meta-analysis
Source: PLoS One. 2019 Apr 9;14(4):e0214738. doi: 10.1371/journal.pone.0214738 (PMC6456231; doi:10.1371/journal.pone.0214738)

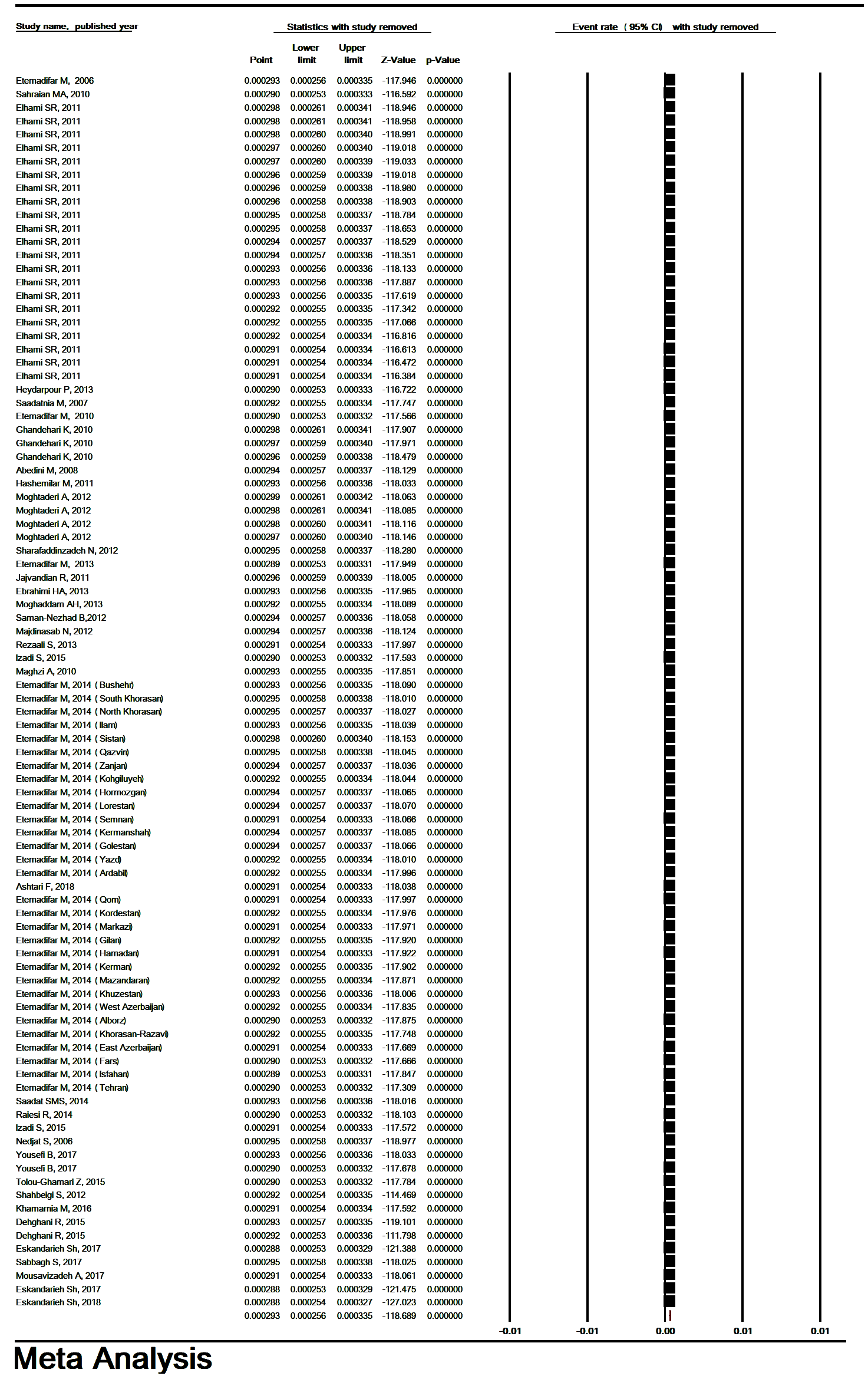

Supplement: S1 Fig — (TIF) [file pone.0214738.s004.tif]

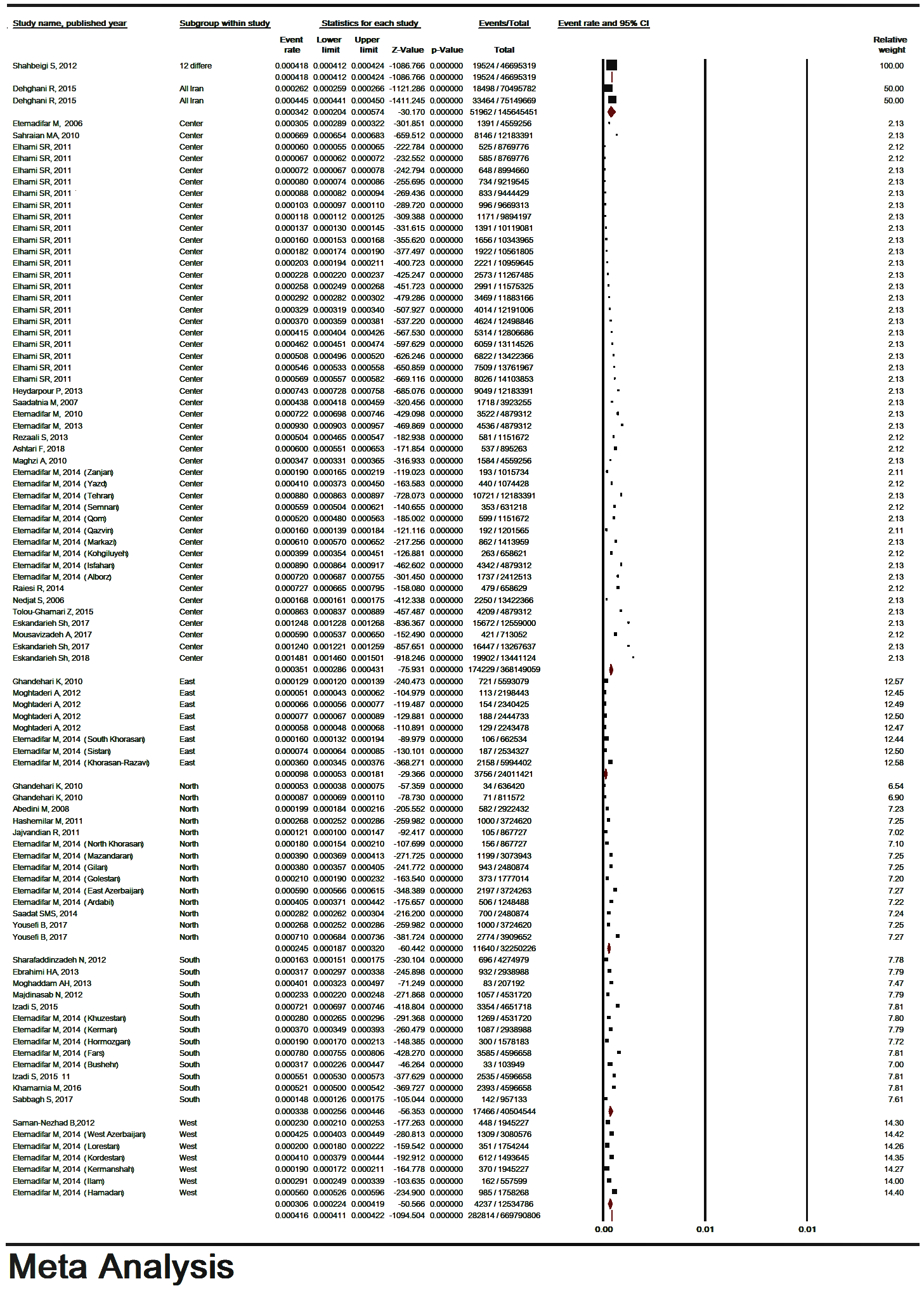

Supplement: S2 Fig — (TIF) [file pone.0214738.s005.tif]

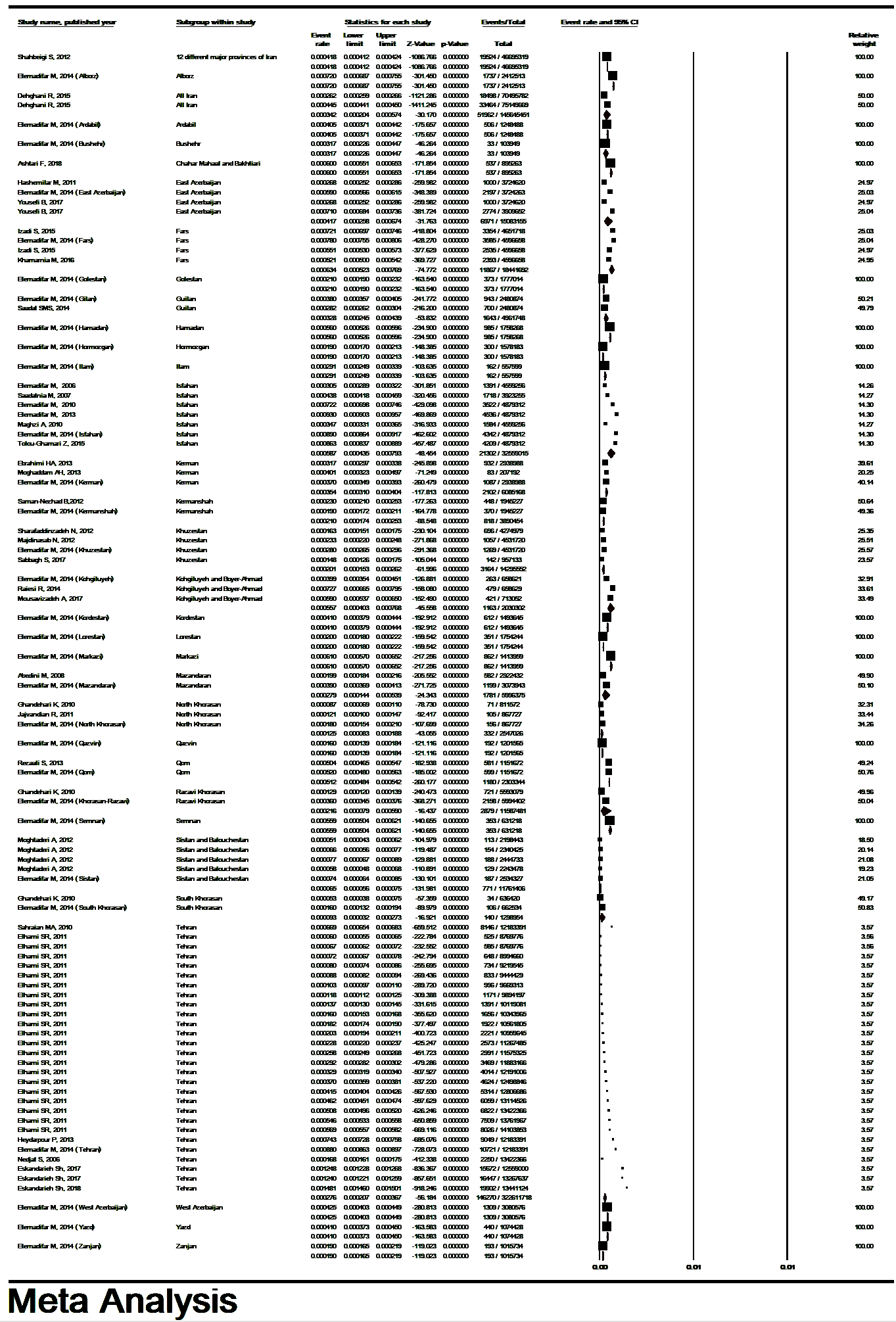

Supplement: S3 Fig — (TIF) [file pone.0214738.s006.tif]

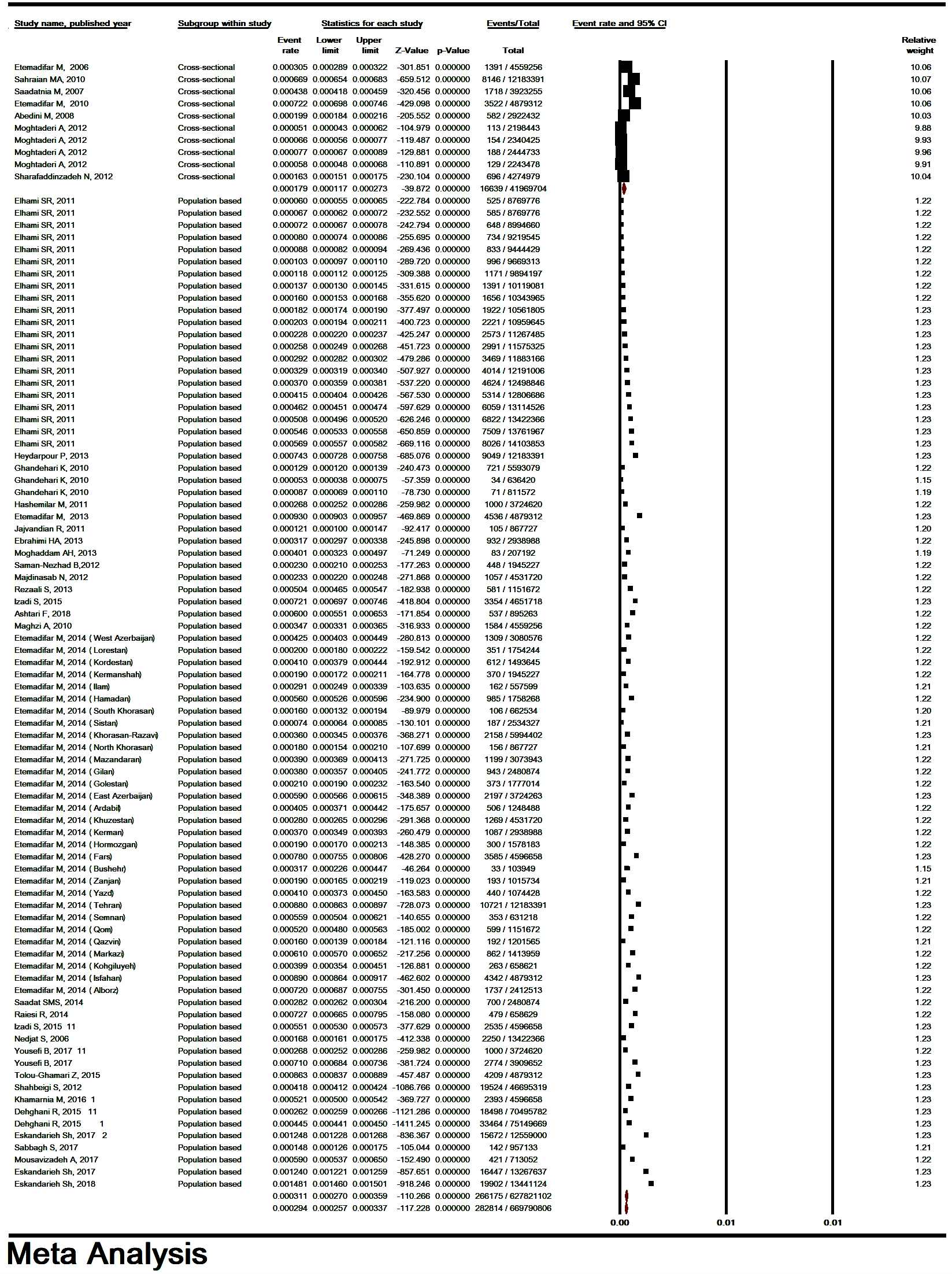

Supplement: S4 Fig — (TIF) [file pone.0214738.s007.tif]

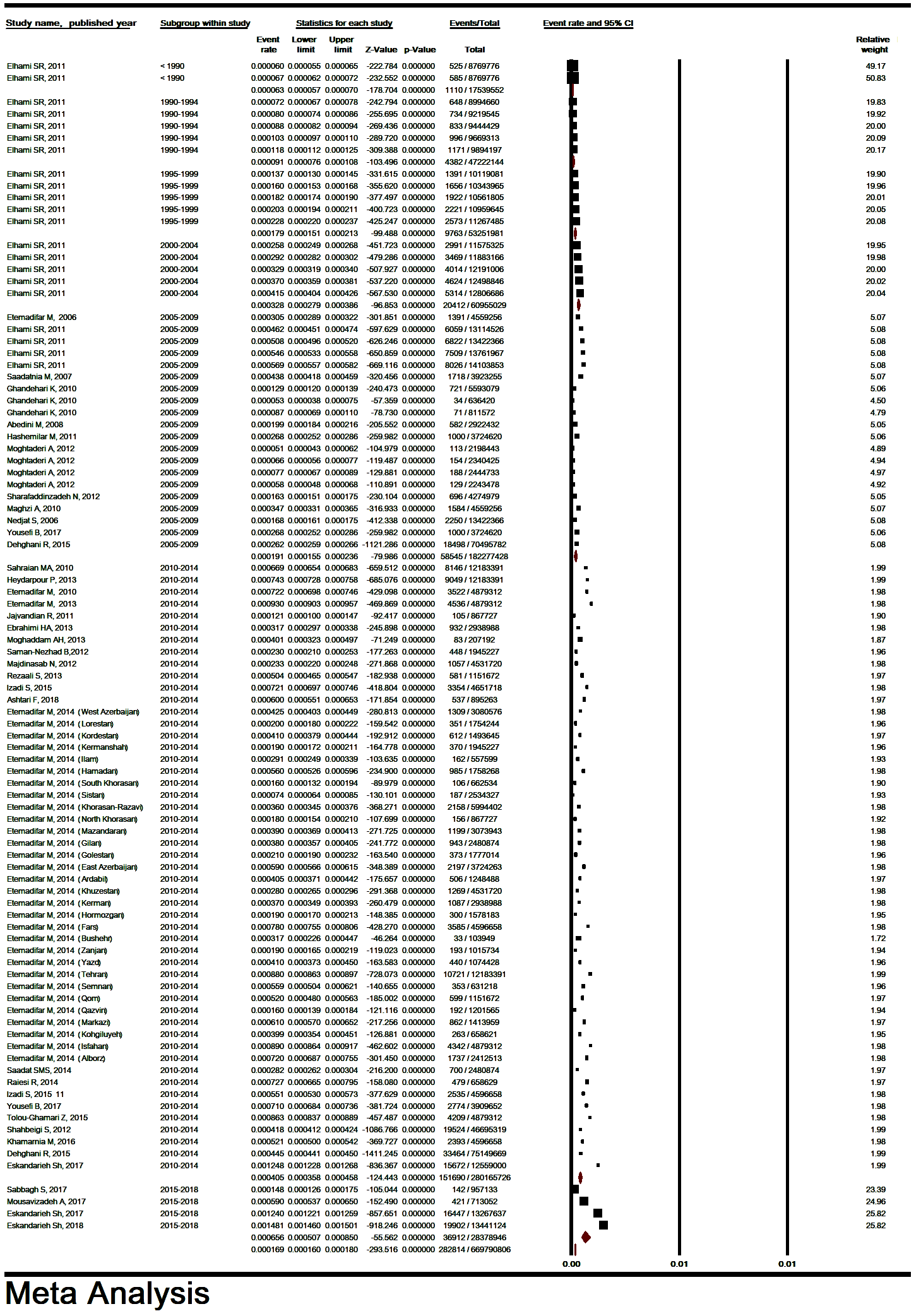

Supplement: S5 Fig — (TIF) [file pone.0214738.s008.tif]

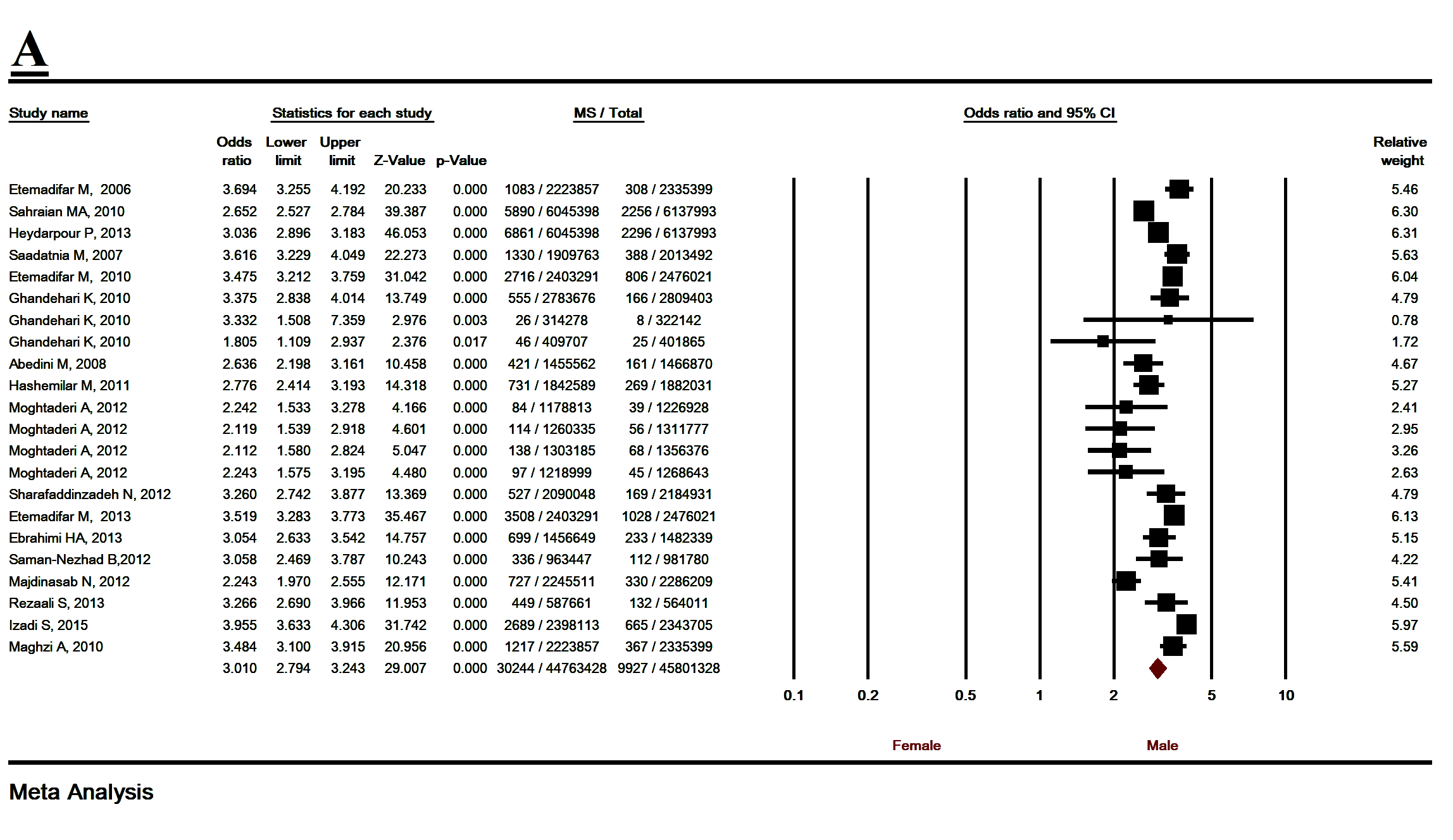

Supplement: S6 Fig — The OR female to male of MS prevalence (A) and incidence (B). (TIF) [file pone.0214738.s009.tif]

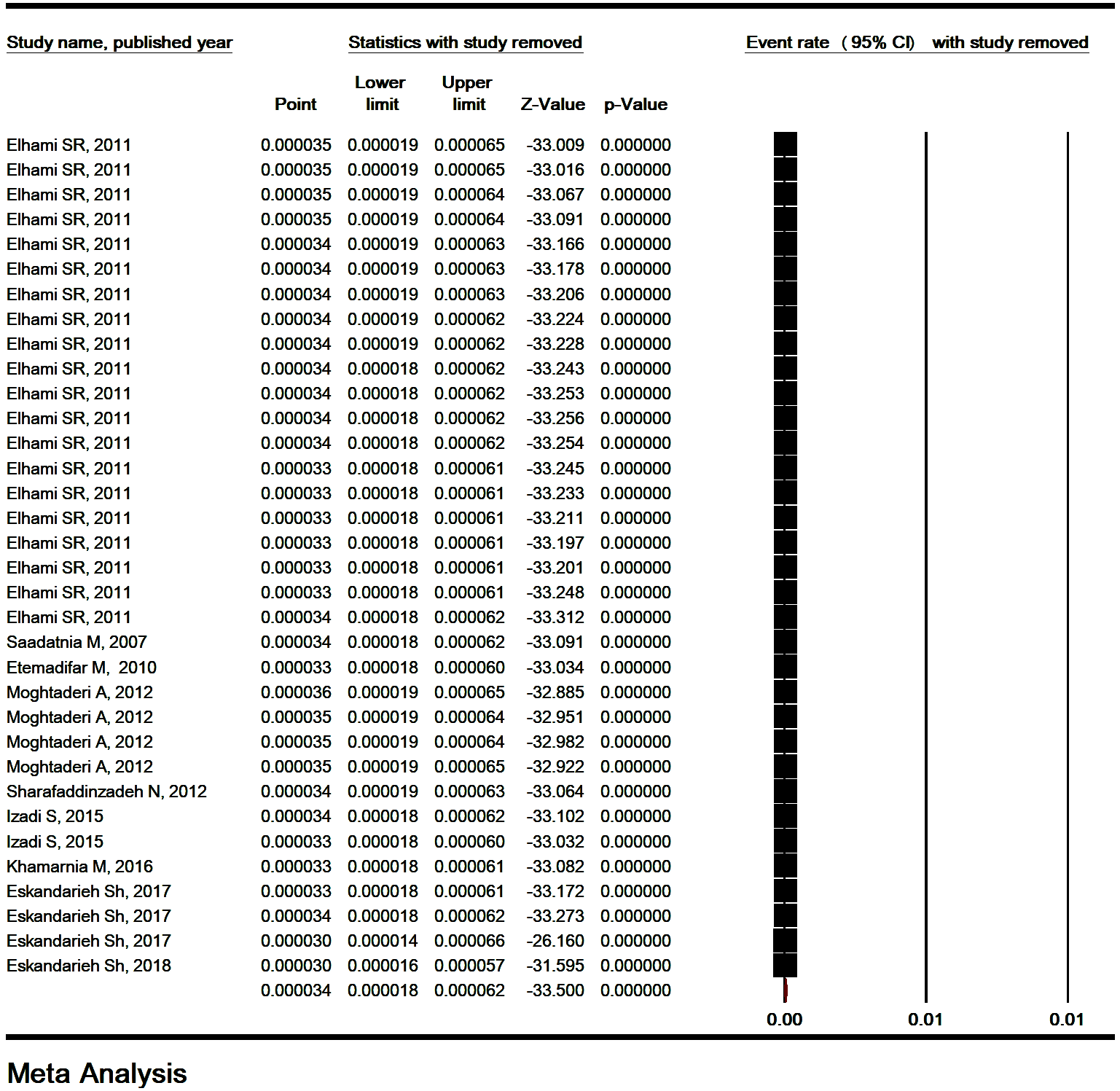

Supplement: S7 Fig — (TIF) [file pone.0214738.s010.tif]

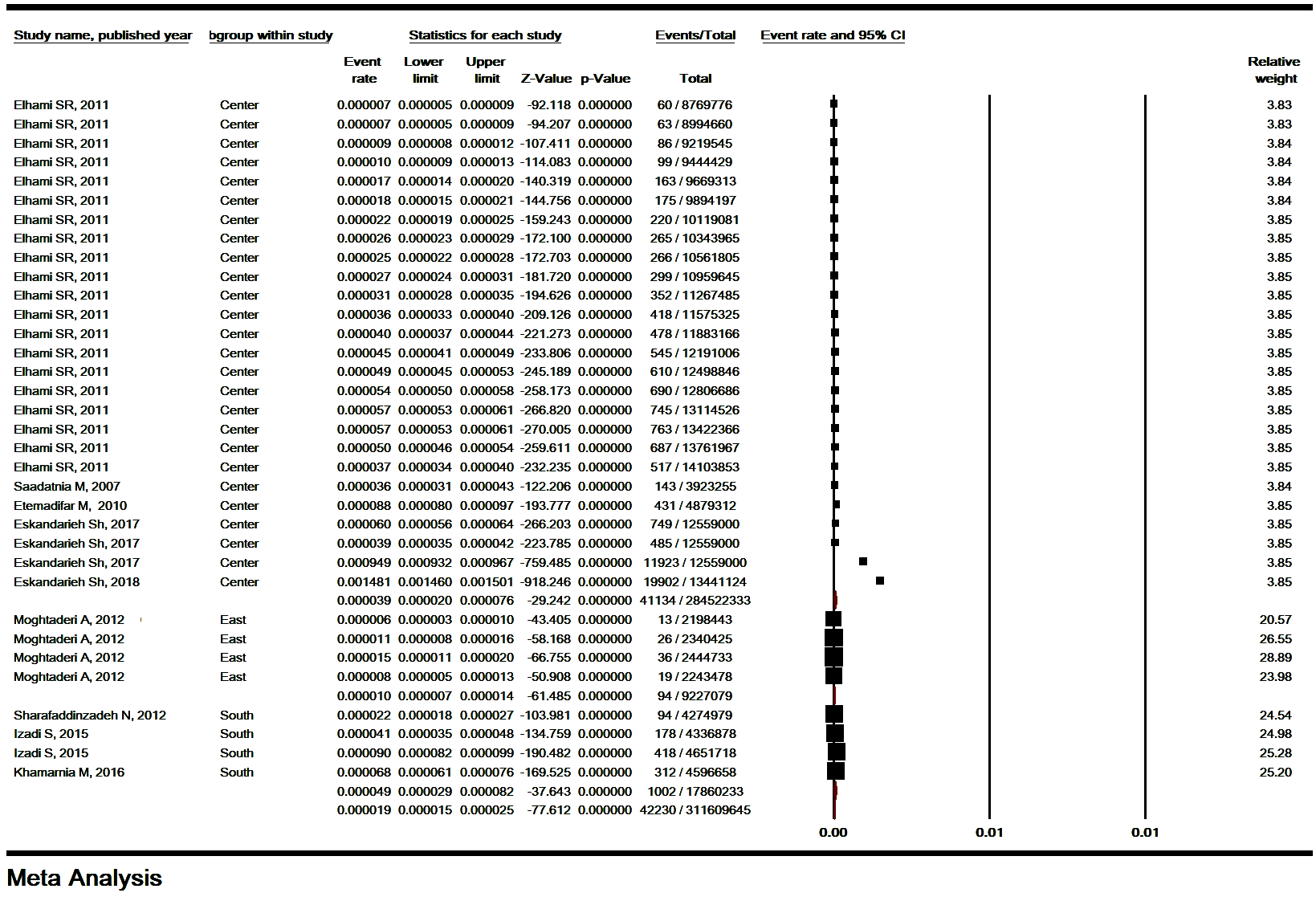

Supplement: S8 Fig — (TIF) [file pone.0214738.s011.tif]

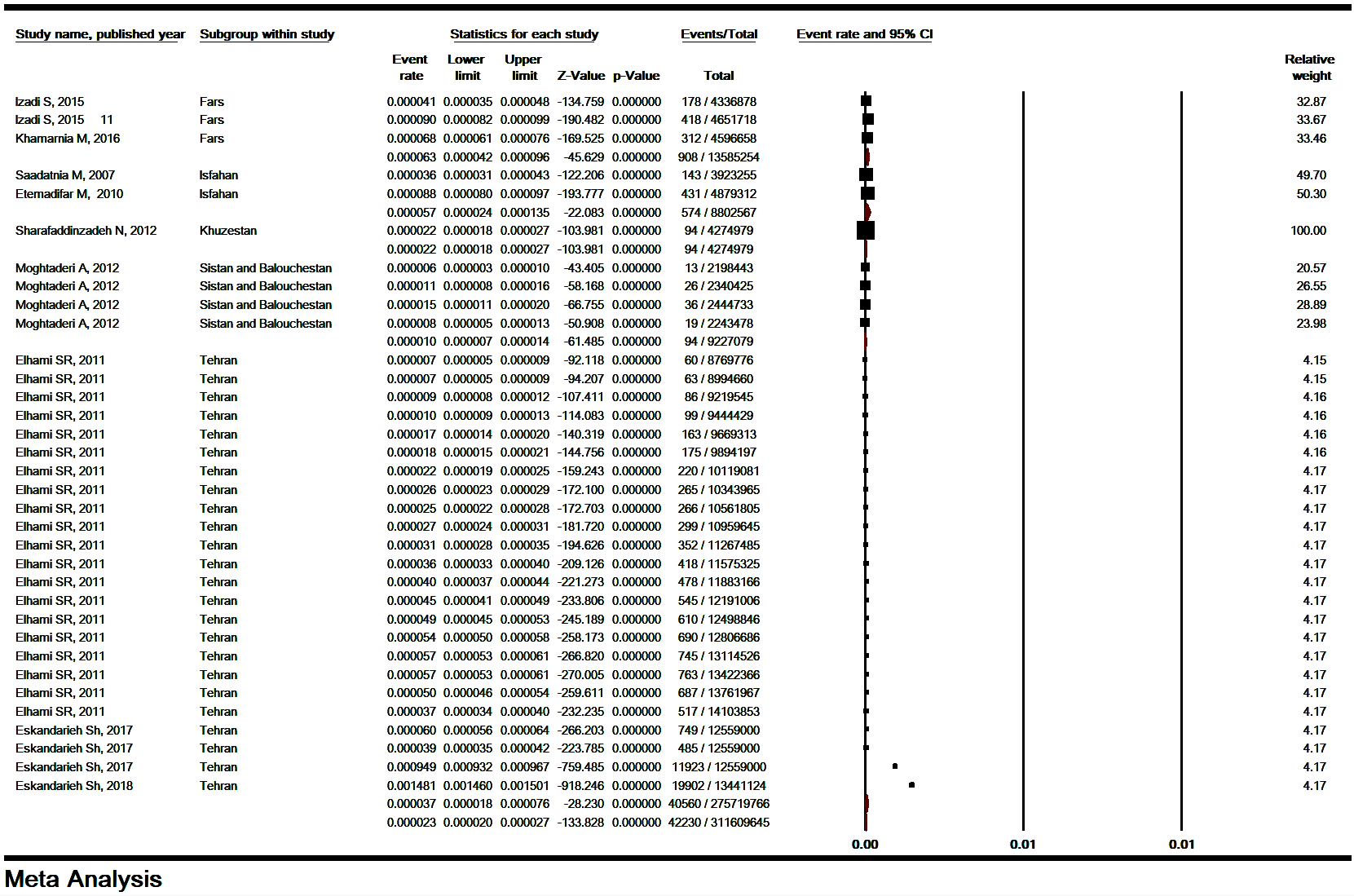

Supplement: S9 Fig — (TIF) [file pone.0214738.s012.tif]

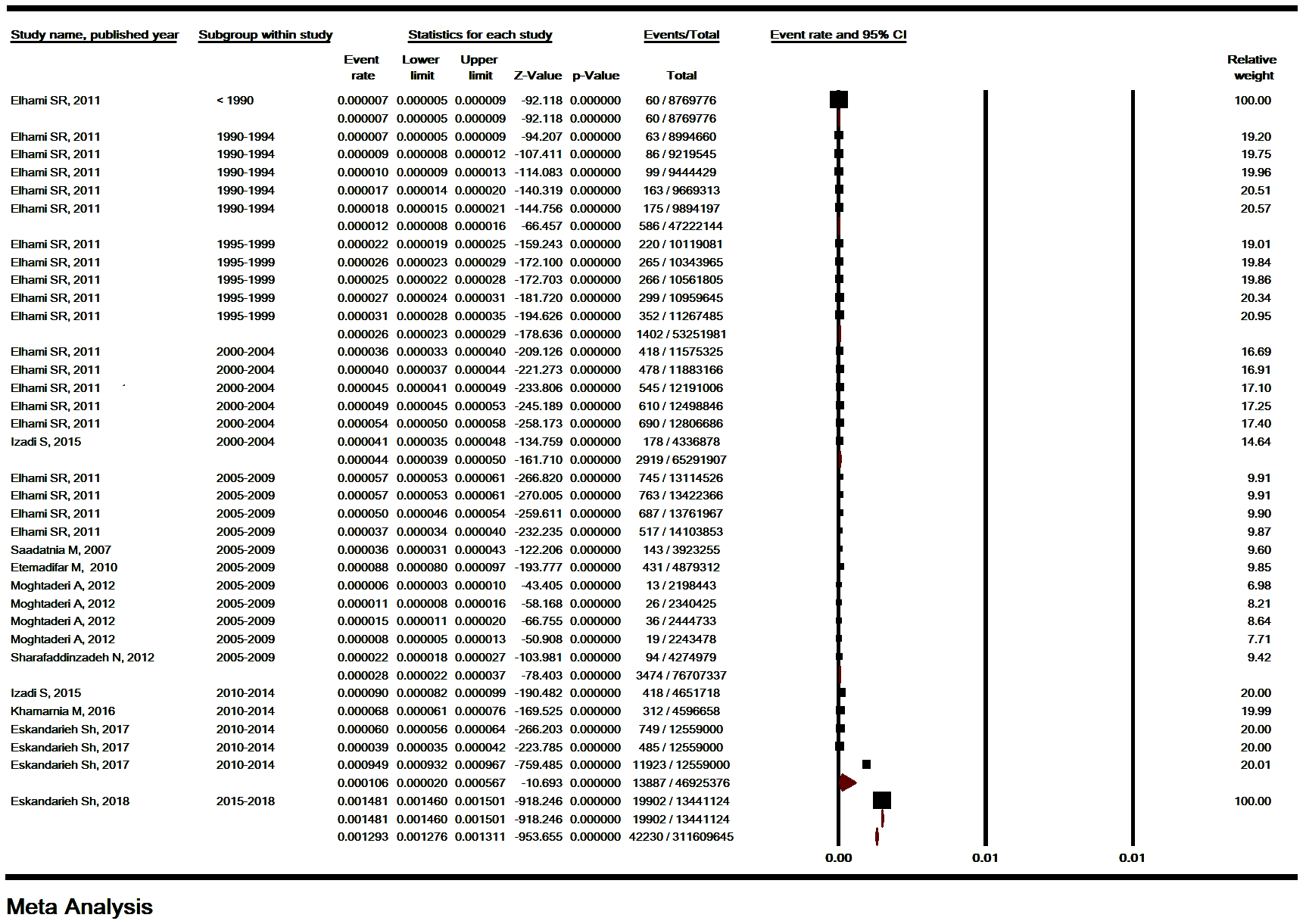

Supplement: S10 Fig — (TIF) [file pone.0214738.s013.tif]

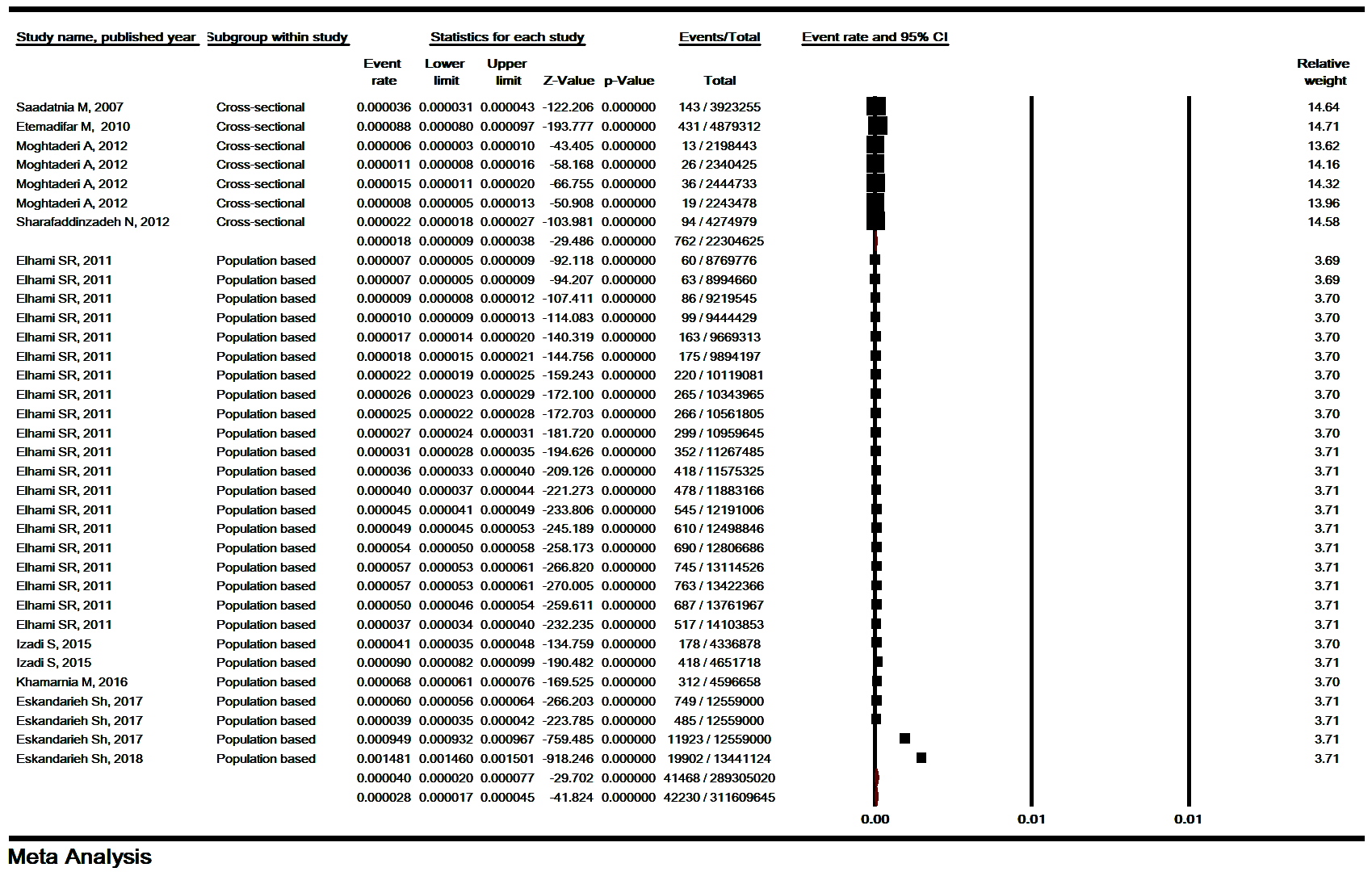

Supplement: S11 Fig — (TIF) [file pone.0214738.s014.tif]

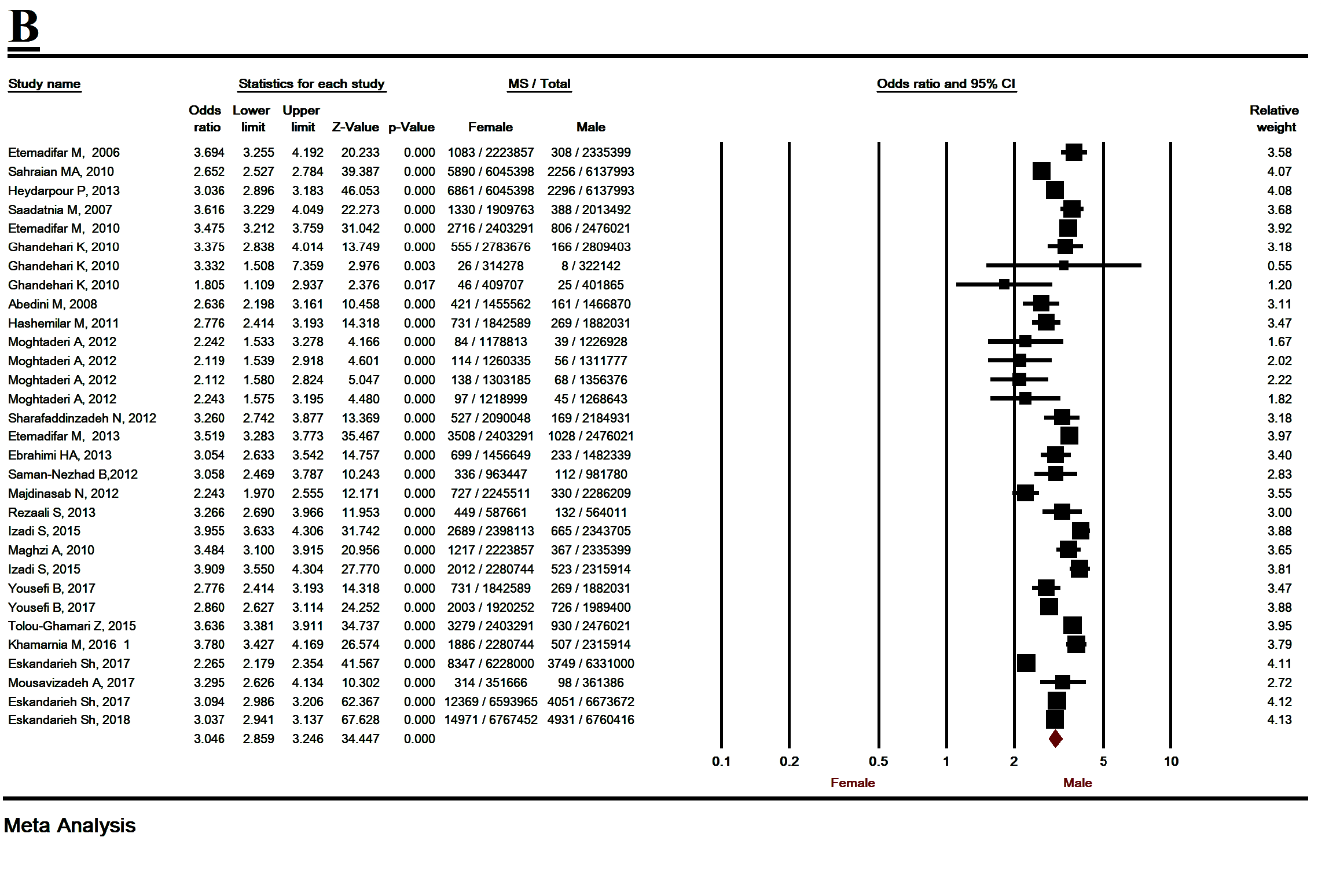

Supplement: S12 Fig — (TIF) [file pone.0214738.s015.tif]

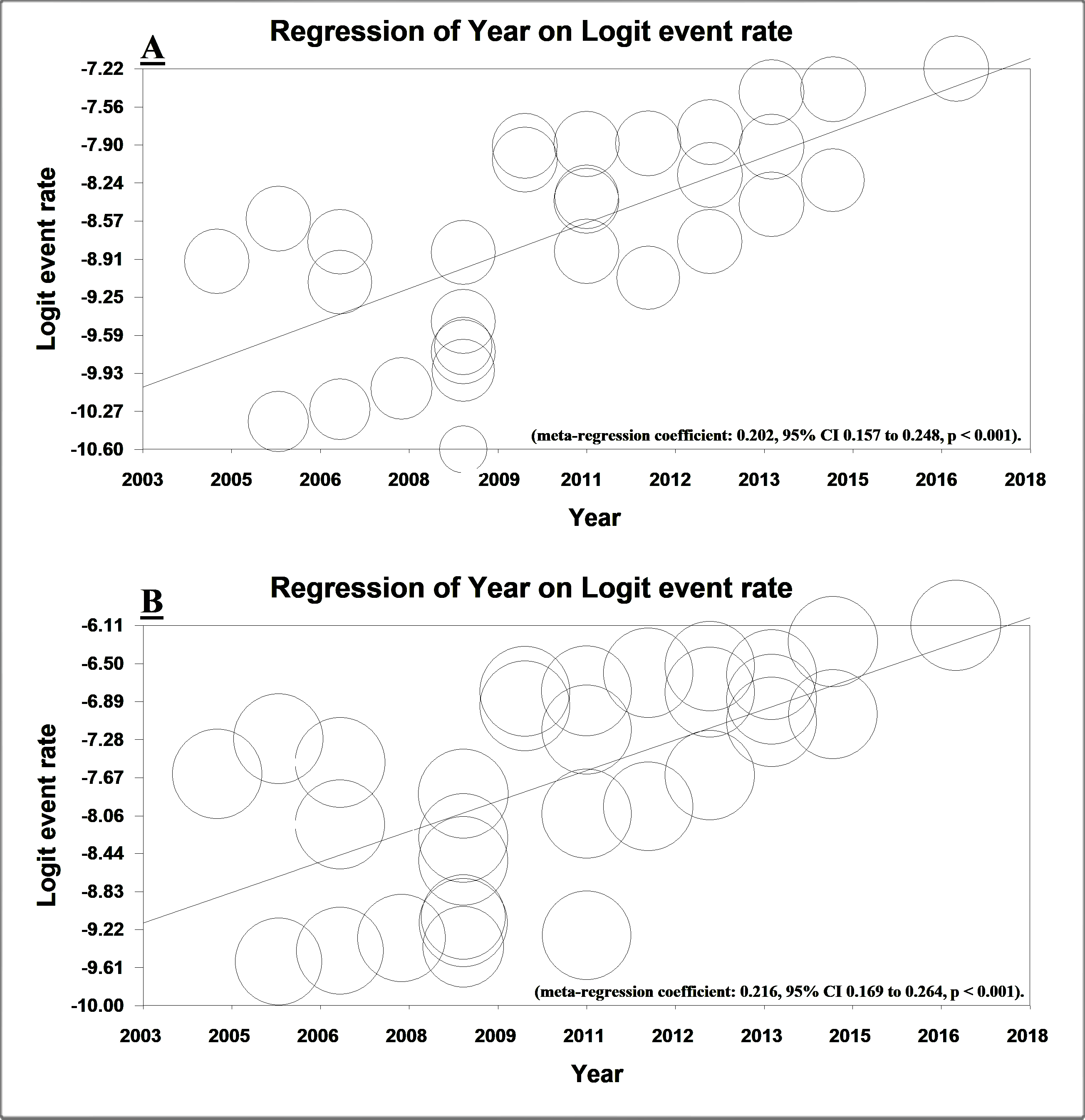

Supplement: S13 Fig — Prevalence of Multiple Sclerosis in Iran in terms of men (A) and women (B). (TIF) [file pone.0214738.s016.tif]

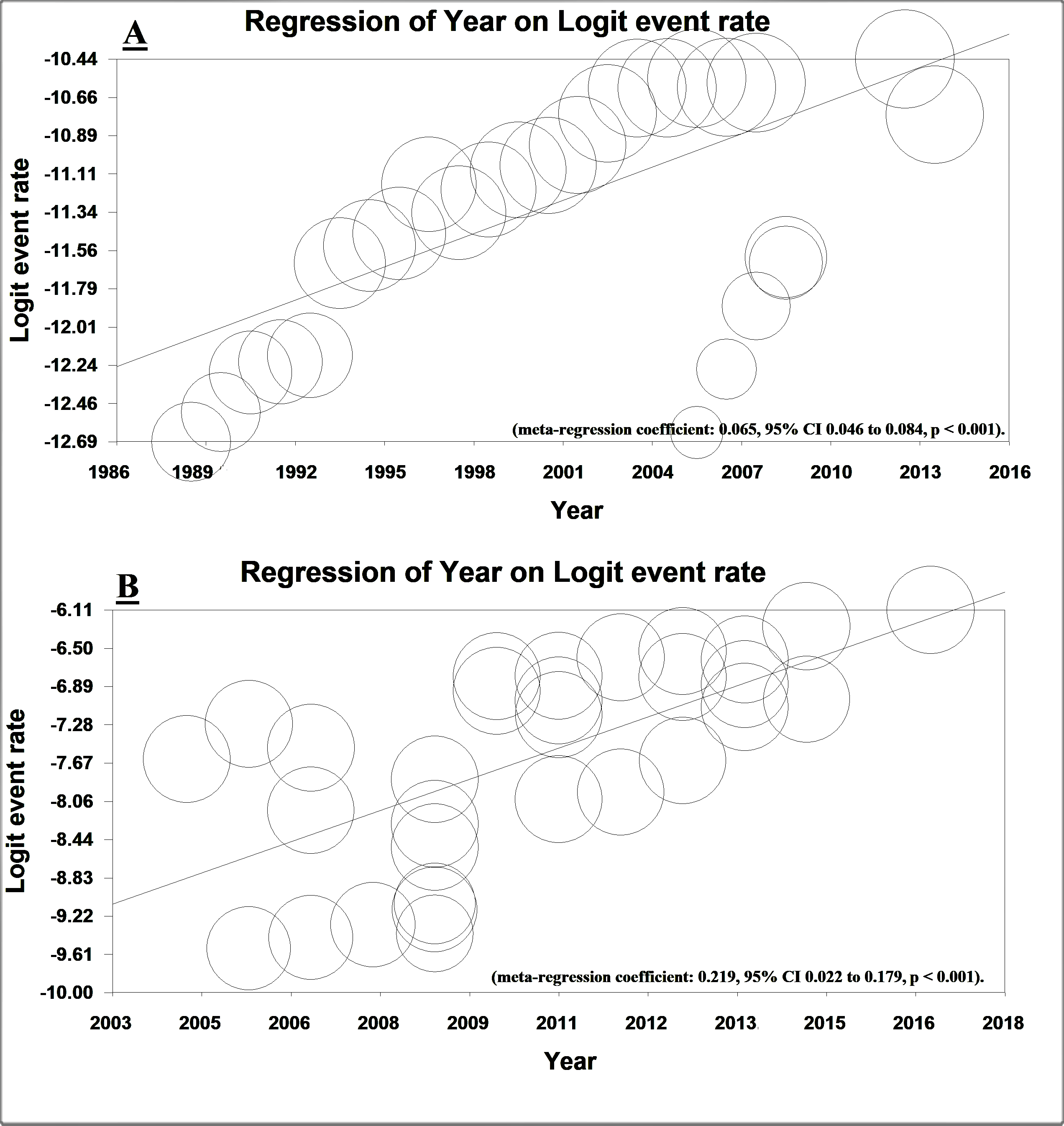

Supplement: S14 Fig — Incidence of Multiple Sclerosis in Iran in terms of men (A) and women (B). (TIF) [file pone.0214738.s017.tif]

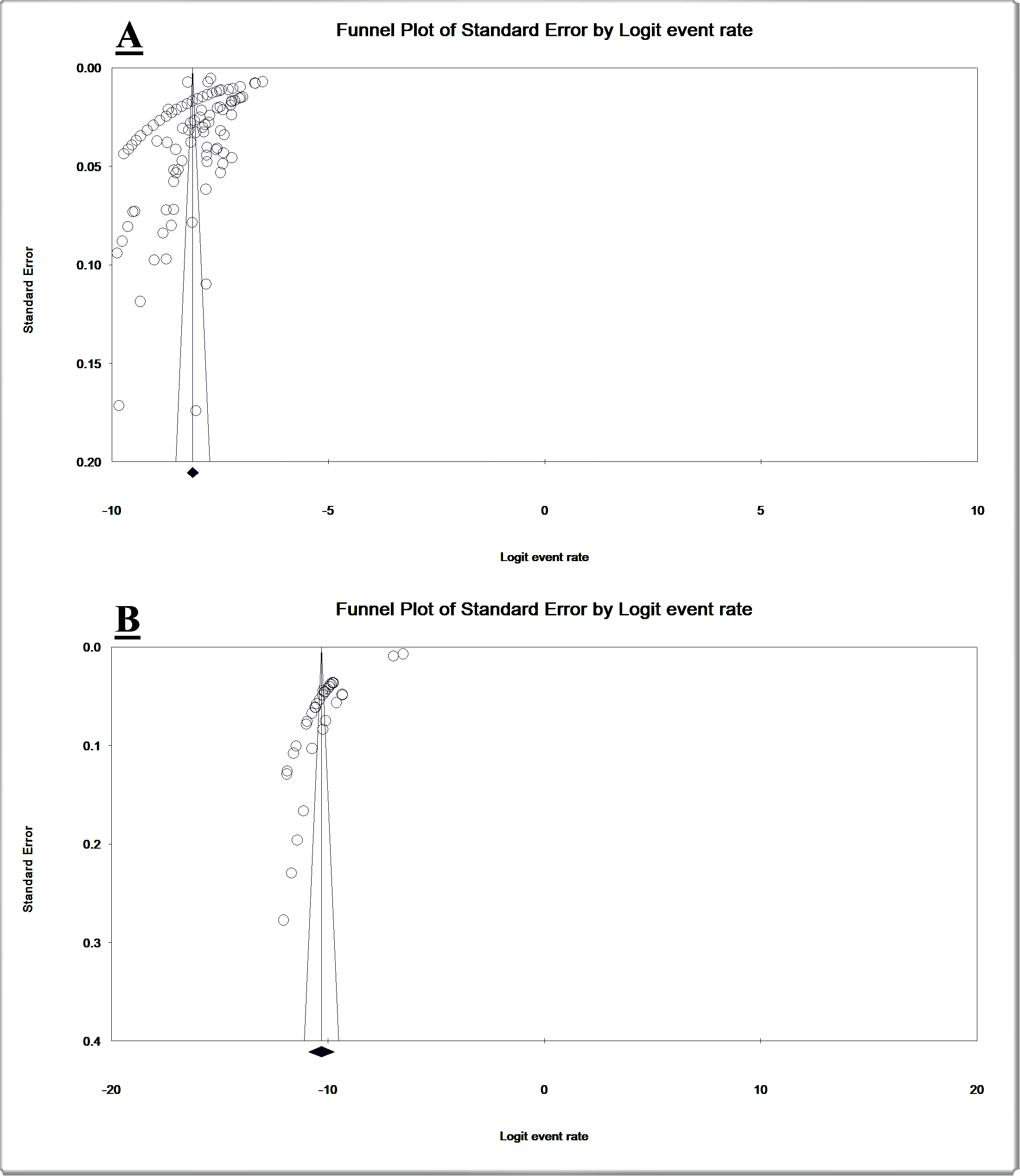

Supplement: S15 Fig — Publication bias for prevalence studies (A) and update (B) multiple sclerosis in Iran. (TIF) [file pone.0214738.s018.tif]
